# Identification of an integrated stress and growth response signaling switch that directs vertebrate intestinal regeneration

**DOI:** 10.1186/s12864-021-08226-5

**Published:** 2022-01-04

**Authors:** Aundrea K. Westfall, Blair W. Perry, Abu H. M. Kamal, Nicole R. Hales, Jarren C. Kay, Madhab Sapkota, Drew R. Schield, Mark W. Pellegrino, Stephen M. Secor, Saiful M. Chowdhury, Todd A. Castoe

**Affiliations:** 1 Department of Biology, University of Texas at Arlington, Arlington, TX USA; 2 Advanced Technology Cores, Baylor College of Medicine, Houston, TX USA; 3 Department of Chemistry and Biochemistry, University of Texas at Arlington, Arlington, TX USA; 4 Department of Research Development and Commercialization, University of North Texas Health Science Center, Fort Worth, TX USA; 5 Department of Biological Sciences, University of Alabama, Tuscaloosa, AL USA; 6 Department of Internal Medicine, University of Texas Southwestern Medical Center, Dallas, TX USA; 7 Department of Ecology and Evolutionary Biology, University of Colorado Boulder, Boulder, CO USA

**Keywords:** mTOR, NRF2, Phosphoproteomics, RNAseq, Unfolded protein response

## Abstract

**Background:**

Snakes exhibit extreme intestinal regeneration following months-long fasts that involves unparalleled increases in metabolism, function, and tissue growth, but the specific molecular control of this process is unknown. Understanding the mechanisms that coordinate these regenerative phenotypes provides valuable opportunities to understand critical pathways that may control vertebrate regeneration and novel perspectives on vertebrate regenerative capacities.

**Results:**

Here, we integrate a comprehensive set of phenotypic, transcriptomic, proteomic, and phosphoproteomic data from boa constrictors to identify the mechanisms that orchestrate shifts in metabolism, nutrient uptake, and cellular stress to direct phases of the regenerative response. We identify specific temporal patterns of metabolic, stress response, and growth pathway activation that direct regeneration and provide evidence for multiple key central regulatory molecules kinases that integrate these signals, including major conserved pathways like mTOR signaling and the unfolded protein response.

**Conclusion:**

Collectively, our results identify a novel switch-like role of stress responses in intestinal regeneration that forms a primary regulatory hub facilitating organ regeneration and could point to potential pathways to understand regenerative capacity in vertebrates.

**Supplementary Information:**

The online version contains supplementary material available at 10.1186/s12864-021-08226-5.

## Background

Vertebrates have extensive variation in regenerative capacity, and identifying conserved vertebrate regenerative mechanisms holds promise both for developing novel strategies for therapeutic regeneration of tissues following injury or disease and for understanding and treating cancer. Non-traditional model systems that possess extreme regenerative phenotypes are particularly powerful for identifying the molecular basis of such phenotypes and may provide valuable insight into regenerative capacities and pathways in vertebrates. Most known examples of vertebrate regeneration involve regrowth of appendages, including fin regeneration in fish [1–3], limb and tail regeneration in salamanders [4], and tail regeneration in some reptile groups [5–7]. Fewer examples exist for vertebrate regeneration of complex organ tissues: salamanders and fish are both capable of regrowing brain and cardiac tissue [8–12], and many vertebrates regenerate liver tissue [13]. Snakes, however, represent an intriguing outlier among vertebrates; upon feeding, they undergo extreme regenerative organ growth in multiple organ systems [14–18] that is unparalleled in magnitude. This regenerative capacity in snakes offers a unique perspective on how particular lineages might exploit conserved signaling pathways to achieve unique regenerative phenotypes and thereby reveal novel therapeutic targets relevant to humans.

Multiple heavily bodied lineages of snakes (including boas, pythons, and rattlesnakes) that experience months-long fasts between meals have evolved the ability to downregulate and atrophy energetically costly digestive organs during fasting, only to subsequently regenerate organ tissue and function at unequaled rates and magnitudes after feeding. For example, within 24 h of consuming a meal, the Burmese python (*Python bivittatus*), increases its small intestine wet mass two-fold, intestinal mucosa thickness three-fold, and intestinal microvillus length five-fold [19–23]. Physiological activity simultaneously increases, including a 44-fold increase in metabolic rate and 20-fold increase in intestinal nutrient transport [15, 16, 22–25]. Previous studies characterized changes in gene expression associated with intestinal regenerative growth in snakes and implicated the involvement of several conserved growth and stress response pathways that function in tissue growth in other vertebrates [16–18]. One study on python intestinal growth hypothesized that growth factors and metabolic stress drive interactions among cell junction and growth signaling, stress response, and DNA damage response pathways to coordinate post-prandial regenerative growth [17], and a follow-up study provided further evidence for these pathways and identified a shared suite of mechanisms that appears to regulate regenerative responses across multiple snake species [18]. The precise molecular mechanisms, pathway interactions, and timing of activation among these mechanisms and pathways remain poorly understood. Furthermore, there is no clear indication of what precise signaling steps or regulatory molecules coordinate major shifts in the regenerative response.

To address these gaps in our knowledge and advance models of vertebrate regeneration, we integrate multiple “omics” datasets and physiological data from a heavy bodied, infrequently feeding snake, the boa﻿ con﻿s﻿﻿tric﻿tor (*Boa constrictor*), that experiences post-feeding regenerative intestinal growth [22]. We sample small intestine from these snakes over the course of a time series that spans from fasting to 6 days post-feeding (dpf) states to understand the precise timing and integration of signaling networks, identify regulatory molecules that drive these signaling cascades, and link these to changes in cell and tissue physiology during post-prandial intestinal regeneration. To understand the processes that initiate the regenerative growth response, we identify mechanisms that respond to the massive nutrient uptake which precedes most tissue growth and generates extreme metabolic stress. We demonstrate distinct regulatory relationships among multiple growth and stress response pathways and define a model of the regenerative mechanism that includes detailed interactions between broadly conserved vertebrate signaling pathways that underlie the regenerative process. Our results highlight, for the first time, key regulatory kinases that coordinate the response, by integrating signaling from stress response pathways (e.g., NRF2 signaling, unfolded protein response (UPR), 14–3-3 signaling) and nutrient-sensing and growth pathways (e.g., mTOR, PI3K/AKT). These new findings highlight the previously underappreciated interaction between growth and stress responses in the context of major tissue remodeling and regeneration and identify potential molecules that hold promise for therapeutic control of regenerative responses.

## Results

### Feeding triggers rapid, extreme changes in intestinal form and function

**Fig. 1:**
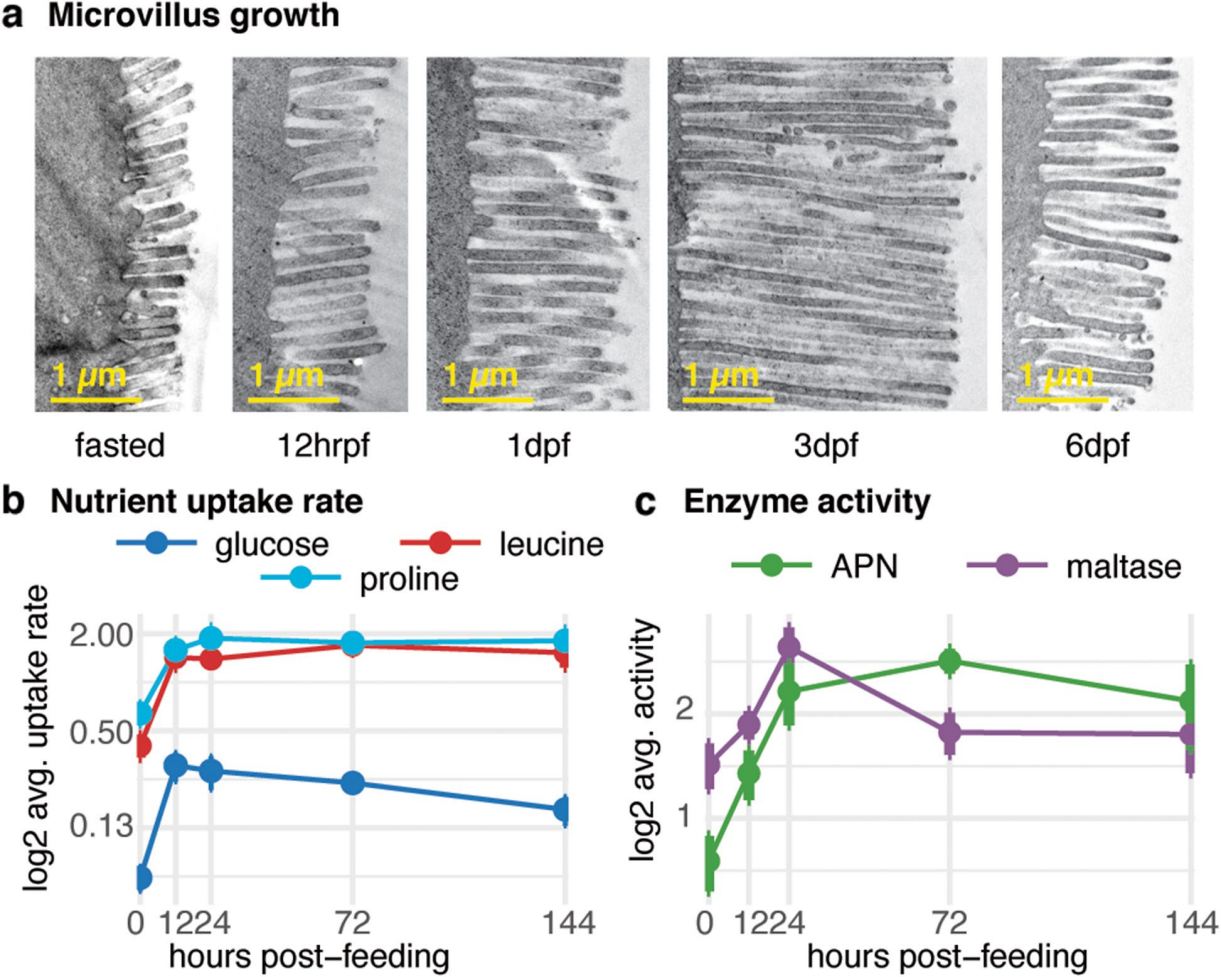
Massive increases in intestinal form and function following feeding in the *boa constrictor*. **a** Electron microscope images of microvilli at different stages of digestion show the extreme shifts in intestinal morphology following a meal. **b** Nutrient uptake rate and **c** small intestine enzyme activity throughout digestion. Whiskers indicate standard error

We sampled small intestine tissues from 3 to 5 snakes at each of five time points: fasted, 12 h post-feeding (hrpf), 1dpf, 3dpf, and 6dpf. Within 6 days following feeding, the small intestine wet mass increases by over 100%, mucosal width doubles, and enterocyte volume doubles by 6dpf (Fig. S1a-c). At the ultrastructural level, there is a 10-fold increase in intestinal epithelial microvillus length by 3dpf (Fig. 1a). Changes in intestinal form coincide with increases in intestinal performance: nutrient uptake rates for proline, leucine, and glucose increase significantly between three- and five-fold within 12 h (Fig. 1b), and intestinal enzyme activity for aminopeptidase N (APN) and maltase increases two- to four- fold (Fig. 1c). Nutrient uptake and enzyme activity capacities significantly (*p* < 0.05) increase five- to seven-fold by 12hrpf (Fig. S1d-e).

**Fig. 2:**
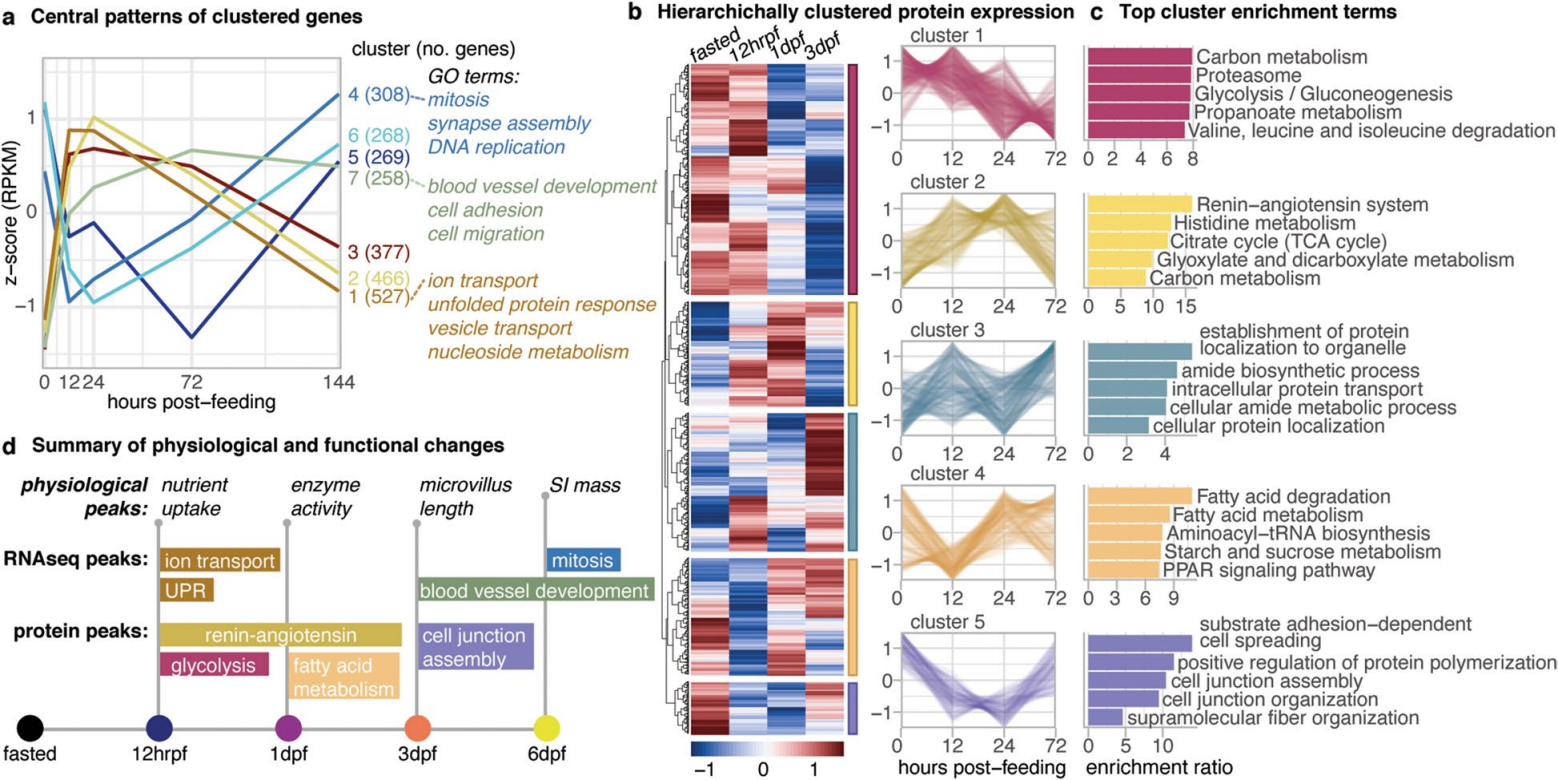
Temporal activity and gene and protein expression supports extreme physiological shifts. **a** Differentially expressed genes and cluster into discrete patterns with specific GO Term functional enrichment throughout the time series. **b** Heatmap of average protein quantitation, scaled by row and hierarchically clustered. Line plots reproduce patterns of protein expression in each cluster. **c** Top five enrichment terms for GO biological process (clusters 1 and 3) or KEGG pathways (clusters 2, 4, and 5) for proteins in each cluster. **d** Summary of major enriched functions aligned with peaks in measured physiological changes throughout the time series

Large shifts in gene expression accompany the complete remodeling of intestinal architecture and massive increase in function, with 1833 genes differentially expressed between fasted and 12hrpf (Table S1). We clustered gene expression profiles over time into seven discrete patterns (Fig. 2a). The two largest gene clusters both exhibit rapid upregulation: cluster 1 (527 genes) is characterized by sustained high expression from 12hrpf to 1dpf, and cluster 2 (466 genes) by peak upregulation at 1dpf. Functional enrichment terms associated with genes in cluster 1 include ion transport, the UPR, vesicle transport, nucleoside metabolism, and carbohydrate transmembrane transport, while cluster 2 genes are enriched for phosphatase binding, RNA polymerase II DNA binding, and coenzyme binding (Figs. 2a, S2a-c). In contrast, clusters 4 (308 genes) and 6 (268 genes) genes are significantly downregulated over the first 12hrpf and 1dpf, respectively, and both recover signaling and are upregulated above fasted levels by 6dpf. Enriched functional terms for these genes include regulation of cytokinetic process, mitotic sister chromatic segregation, and centromere complex assembly (Figs. 2a, S2d-f).

To complement our physiological and transcriptomic analyses, we quantified protein and phosphoprotein expression from a subset of samples (Table S3). Our quantitative proteomic analyses identify 922 proteins with high confidence, 286 of which are also present among differentially expressed transcripts, and we clustered proteins based on expression (Fig. 2b). Cluster 1 (331 proteins) is highly expressed through fasted and 12hrpf before steadily declining in later time points, with top enrichment for functions in metabolism, proteasomes, and glycolysis and gluconeogenesis (Fig. 2c). This is consistent with RNAseq predictions of high metabolic activity and protein degradation linked to stress in the early post-feeding response (Fig. 2a). Cluster 2 (198 proteins) is enriched for terms related to protein localization and cellular transport (Fig. 2c), with two separate peaks of expression at 12hrpf and 3dpf. The 168 proteins in Cluster 3 decrease at 12hrpf but increase thereafter, and they encompass functions related to fatty acid degradation and metabolism, starch and sucrose metabolism, and PPAR signaling (Fig. 2c). Cluster 4 (150 proteins) exhibits high levels by 1dpf and is enriched for the renin-angiotensin system, TCA cycle, and multiple metabolic pathways (Fig. 2c). In contrast, Cluster 5 (75 proteins) shows the inverse pattern of low levels at 1dpf and is enriched for cell junction assembly and organization, protein polymerization, and substrate adhesion-dependent cell spreading (Fig. 2c). Collectively, quantitative proteomic results confirm key functional inferences from RNAseq data and highlight evidence for responses to high metabolic activity and stress early in the post-feeding response. Observations of high levels of cell adhesion and junction assembly proteins and rebounding increases in the quantity of transport proteins at 3dpf suggest a second wave of major shifts in cellular function linked to decreased stress and preparation for renewed cell growth and proliferation pathways during the later phase of regeneration.

Overall, both RNA and protein quantification identify an early wave (from fasted through 1dpf) of transport, stress response, and metabolic functions. Signals at 3dpf and 6dpf highlight major changes to tissue form as cell junction organization, blood vessel development, and mitosis increase. These patterns correspond to changes measured in the intestine, where we find early peaks in uptake and metabolic activity while microvillus, cell, and tissue growth do not reach their peak levels until 3dpf and 6dpf.

### Stress and growth responses modulate intestinal regenerative growth

**Fig. 3:**
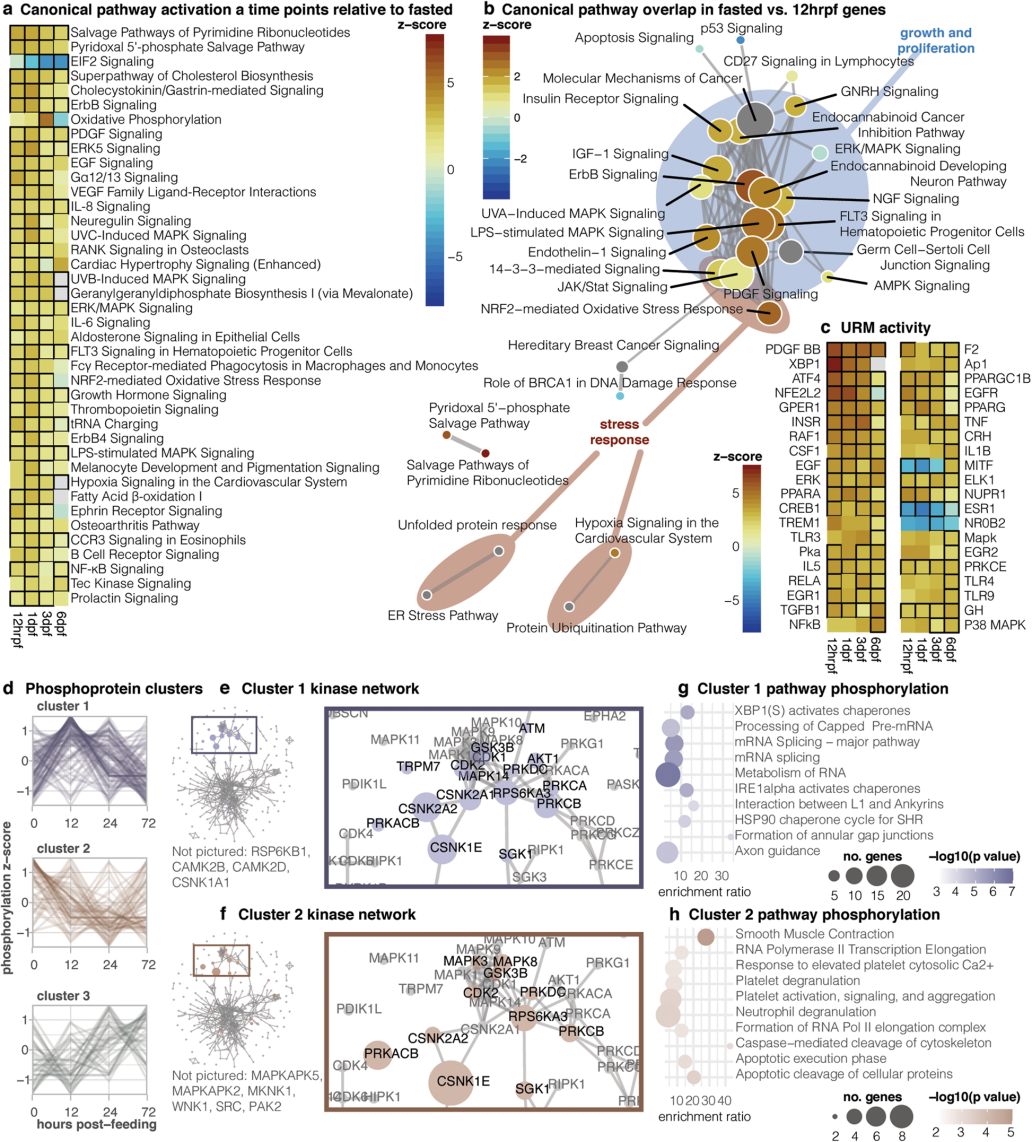
Key signaling pathways and regulatory molecules drive boa intestinal regeneration. **a** Heatmap of canonical pathway activation and **b** URM activity from IPA, generated by each time point relative to fasted. **c** Overlapping pathway activity from genes significantly differentially expressed between fasted and 12hrpf. Circle size indicates “indegree”, or centralization within the network. **d** Phosphorylation patterns for three clusters of quantified phosphoproteins. **e**, **f** Kinase networks, indicating enrichment of specific kinases for clusters 1 and 2, with magnification of major groups of active kinases. **g**, **h** Top enriched Reactome pathways from phosphoprotein clusters

To further investigate the functional significance of differentially expressed genes, we conducted Upstream Regulatory Molecule (URM) and Canonical Pathway activity predictions using Ingenuity Pathway Analysis (IPA; Fig. 3a-c). We also inferred the downstream effects of pathway activation patterns with the molecule activity prediction in IPA. Canonical pathways with significant activation changes group into major functional categories: uptake and metabolism, stress response, cell death, cell cycle and DNA damage response, and growth and proliferation. We find significant activity changes from three major nutrient sensing and signaling pathways throughout the post-feeding time series: Insulin Receptor Signaling, IGF-1 Signaling, and mTOR Signaling (Figs. 3a, S3, S4, S5). Two transport-associated pathways exhibit activation changes: PPARα/RXRα Signaling at 12hrpf compared to fasted and Aldosterone Signaling in Epithelial Cells at 1dpf compared to fasted (Figs. 3a, S3, S6). This is also consistent with a spike in PPAR signaling inferred by protein quantification results (Fig. 2c). Other major metabolic pathways active during the feeding response include the Superpathway of Cholesterol Biosynthesis, Oxidative Phosphorylation, and Fatty Acid β-oxidation I (Fig. 3a).

Concurrent with metabolic pathway activation, we identify broad activation of multiple stress response and stress-activated pathways, such as ERK5 Signaling, IL-8 Signaling, IL-6 Signaling, the NRF2-mediated Oxidative Stress Response, Hypoxia Signaling in the Cardiovascular System, and NF-κB signaling (Fig. 3a). At 12hrpf specifically, other activated or significantly present major stress response pathways are Protein Ubiquitination, the UPR, 14-3-3 Signaling, and AMPK Signaling (Figs. S3, S7). Based on both z-score and *p*-value, the NRF2 stress response is the most strongly upregulated pathway from fasted to 12hrpf (Fig. S8). Molecule activity prediction downstream of UPR activation at 12hrpf suggests suppression of apoptosis (Fig. S9), while inhibition of eIF2 Signaling at 1dpf promotes apoptosis and endoplasmic reticulum (ER) stress response (Fig. S10). Other pathways linked to apoptosis are activated at 12hrpf, including TGF-β Signaling, p38 MAPK Signaling, and Death Receptor Signaling (Fig. S3), as well as a pro-apoptosis branch of p53 Signaling (Fig. S11).

Coinciding with metabolic and stress response pathways are changes in activity of growth pathways that regulate cell death, growth, division, and differentiation (Fig. 3a). PI3K/AKT Signaling significantly increases at 12hrpf compared to fasted (Fig. S3), and IPA infers patterns of molecule activation leading to both increased cell growth and survival and decreased cell cycle progression and cell death. Other major growth and proliferation pathways that upregulate after feeding include ErbB Signaling, EGF Signaling, ERK/MAPK Signaling, and Growth Hormone Signaling (Fig. 3a). For several activated growth and proliferation pathways (e.g., JAK/STAT and PDGF), patterns of activity indicate activation of a branch of the pathway not involved in cell proliferation or even downstream suppression of proliferation (Figs. S12, S13). Many cell cycle-specific pathways are inhibited at 12hrpf (Fig. S14): ATM Signaling, Role of BRCA1 in DNA Damage Response, PTEN Signaling, and Cell Cycle: G2/M DNA Damage Checkpoint Regulation. Additionally, although activation of Cell Cycle Control of Chromosomal Replication is significant but nondirectional (i.e., z-score could not be calculated), downstream molecule activity indicates inhibition of the origin recognition complex and DNA replication (Fig. S15). Downstream from these pathways, IPA predicts inhibition of G1/S phase transition, homologous recombination, DNA repair, checkpoint control, and cell cycle progression.

To investigate whether early stress and growth signaling responses cooperate to regulate tissue growth under stress, we examined the relationships and overlap among pathways with activity changes during the period from fasted to 12hrpf (Fig. 2b). We identify a large cluster of genes shared among pathways involved in growth, differentiation, and proliferation that functionally overlap and interact with the NRF2 and 14-3-3-mediated signaling pathways (Fig. 2b). These findings highlight overlapping signaling connections that mechanistically link stress response pathways (NRF2 and 14-3-3) with growth pathways (including Insulin/mTOR, JAK/Stat, ERK/MapK) that are co-stimulated within 12 h following feeding (Fig. 2b). Overall, canonical pathway analyses highlight an early increase in transport and high-level metabolic pathways followed by a major surge of stress response and cell death mechanisms. These coordinate to suppress cell cycle progression and prevent significant proliferation while the tissue is under extreme metabolic stress, and near the end of the regeneration timeline, these pathways downregulate as cell proliferation and growth resume.

### Identification of essential regulatory molecules and kinases directing regeneration

Based on evidence for the interaction of key stress response and growth pathways, we investigated specific regulatory molecules that might mediate these relationships to control the regenerative response. In analysis of RNAseq data across timepoints, the top upregulated URMs are PGDF BB, a regulator of vascular permeability [26]; XBP1, a high-level regulator of the UPR; ATF4, a transcription factor activated by both mTORC1 and integrated stress response pathways [27]; and *NFE2L2*, a top regulator of the NRF2-Mediated Oxidative Stress Response (Fig. 3c). From fasted to 12hrpf, 78 of 95 target molecules for XBP1 are consistent with activation of XBP1, with top targets including ApoA1 and *HSPA5* (BiP) (Table S2), and 100 of 137 target molecules are consistent with activation of *NFE2L2* (z-score 6.448, *p* < 0.01) (Tables S3). These two URMs are inactivated and inhibited, respectively, by 6dpf. Several growth and proliferation URMs have the highest activity at 3dpf or 6dpf, near the end of the regenerative response: INSR, EGF, ERK, TGFB1, NF-κB, MITF, ELK1, and GH (Fig. 3c). One early activated URM is RAF1, a known oncogene [28, 29] but which is also capable of tumor suppressant activity related to decreased IL-6 and JAK/STAT3 signaling activity [30], and simultaneous IL-6 and STAT3 activation has been shown to promote tumor formation in gastrointestinal tissues [31]. Multiple toll-like receptors (TLRs) – TLR3, TLR4, and TLR9 – are significantly activated at 6dpf (Fig. 3c). TLRs promote proliferation and cell growth in the intestine; for example, TLR4 expression specifically induces EGF programs of proliferation [32]. Although NF-κB pathways are activated from 12hrpf, as a URM NF-κB is only significantly activated at 6dpf, alongside Ap-1. Both are important transcription factors stimulated by TLRs [33].

Our phosphoproteomic analyses identify 222 phosphoproteins with quantitative changes across time points, which we group into four clusters comprising 114, 67, 38, and 3 phosphoproteins, respectively (Fig. 3d). Cluster 1 increases abundance by 12hrpf, indicating an important role for post-translational modifications in early regenerative responses. To understand mechanistic links between phosphoprotein levels and kinases that modulate these responses, we conducted kinase enrichment analyses (Fig. 3d-e). These analyses implicate activity by the kinases CSNK1E, CSNK2A2, PRKCB, RPS6KA3, GSK3β, CSNK2A1, CDK2, and AKT1 (*p* < 0.05) for Cluster 1 (Fig. 3e). Gene set enrichment analyses indicate that these phosphoproteins function in mRNA splicing and processing, chaperone protein regulation, and the IRE1-mediated UPR (Fig. 3g). Cluster 2 proteins are generally dephosphorylated after feeding until 3dpf (Fig. 3d). Like cluster 1, proteins in this cluster are enriched for the kinases CSNK1E, RPS6KA3, and PRKCB, and additionally for CAMK2, PRKACB, MLYK, MAPK8, MKNK1, SGK1, and SRC (Fig. 3f), indicating loss of phosphorylative activity of these kinases. Enrichment analyses place these phosphoproteins in pathways for smooth muscle contraction, platelet activation, and apoptosis (Fig. 3h). The two smallest phosphoprotein clusters both peak at 3dpf (Fig. 3c) and were analyzed together for enrichment. These clusters are also enriched for CSNK1E in addition to ATM, MAPK14, PRKAR2B, and PKD2.

### Temporal coordination of growth and stress response pathway networks

**Fig. 4:**
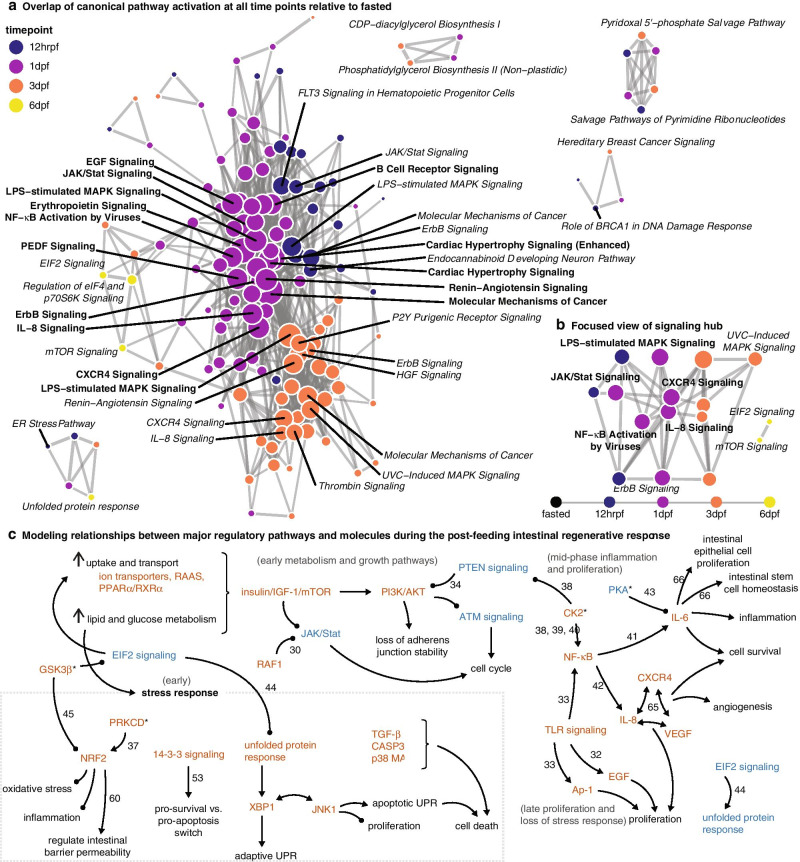
Expanded model summarizing major facets of regenerative growth signaling in *boa constrictor*s after feeding. **a** Pathway overlap of all significantly activated or inhibited canonical pathways from the time series. **b** Close-up view of relationships between major pathways hypothesized to integrate signals from stress response and growth. **c** Summary of early signaling events hypothesized to regulate the early stages of regeneration post-feeding. Molecules and pathways in orange are activated, and in blue are suppressed. Arrowhead ends indicate activation by, and circle ends indicate inhibition by (e.g., PTEN signaling inhibits PI3K/AKT, but PTEN signaling is suppressed, allowing activation of PI3K/AKT). Unless otherwise stated, molecule and pathway activation states and interactions hypothesized are primarily supported by IPA. Activation hypothesized from phosphoproteomic data is indicated by an asterisk (*). Interactions hypothesized from literature are cited

To further investigate the interconnected nature of signaling networks involved in the regenerative response and the temporal dynamics of these interactions, we visualized overlapping pathways activated across all time points (Fig. 4a). All the top centralized nodes (Freeman degree ≥60) except one occur at 1dpf, highlighting 1dpf as a prominent peak of integrated signaling (Fig. 4a). These pathways include JAK/STAT Signaling, Renin-Angiotensin Signaling, Molecular Mechanisms of Cancer, IL-8 Signaling, CXCR4 Signaling, LPS-Stimulated MAPK Signaling, EGF Signaling, PEDF Signaling, NF-κB Activation by Viruses, and B Cell Receptor Signaling (Fig. 4a). Other centralized nodes of pathway activity shift throughout the regenerative response. Although multiple key nutrient sensing and stress response pathways are active at 12hrpf, they do not have high centrality to the broader regenerative network; this suggests that they do not have direct mechanisms of feedback or interaction that link to the central signaling network hub (Fig. 4a). At 3dpf, we find that centralized nodes include tumor-relevant pathways such as UVC-Induced MAPK Signaling and HGF Signaling (Fig. 4a). Collectively, our results identify 1dpf as a major temporal hub of signal activity and integration underlying the regenerative response and highlight the extensive degree of interactions among pathways that coordinate the regenerative response, with important focus on IL-8 Signaling and CXCR4 Signaling at 1dpf and MAPK signaling pathways through 3dpf (Fig. 4b).

### Integrated inference of a novel signaling hypothesis driving intestinal regeneration

Integrated evidence from physiological, RNAseq, proteomic, and phosphoproteomic analyses suggest a model of mechanistic interactions among signal regulators and pathways that modulate regeneration in the snake intestine post-feeding that we organize into three major temporal phases of activity (Fig. 4c). The first largescale change is massive nutrient uptake, triggering response in transporter activity, RAAS activation, and PPARα/RXRα signaling, matched by extreme metabolic activity increase (Fig. 4c). Our results illustrate that nutrient uptake and metabolic shifts activate a major axis of insulin/mTOR signaling and PI3K/AKT/PTEN signaling indicated by increased insulin, IGF-1, and mTOR signaling and suppression of ATM and PTEN signaling. PTEN antagonizes PI3K/AKT activity [34], while mTOR is a negative regulator of ATM signaling, halting cell cycle checkpoints and sensitizing cells to oxidative stress [35] and apoptosis [36] (Fig. 4c). Changes in PI3K/AKT signaling along this axis modulate cell cycle arrest and loss of stability in adherens junctions, while uptake, metabolism, and increased transcriptional activity generate extreme ER and oxidative stress responses. These cellular stress responses are presumably cell-specific and heterogenous throughout the tissue, supported by evidence for both pro-survival and apoptotic signaling regimes moderated by the UPR and 14-3-3 signaling pathways. PRKCD, which we observe at 12hrpf, directly phosphorylates NRF2 to promote antioxidant activity [37], and NRF2 and AMPK signaling accompany other stress responses and likely support pro-survival stress responses in cells that survive, while TGF-β, CASP3, and p38 MAPK regulate the apoptotic responses.

At 12hrpf, our results show that regulatory kinase activity includes phosphorylation by CSNK2A1 and CSNK2A2 (Fig. 3e-f), two key subunits of CK2, a pleiotropic protein kinase that functions in many signaling networks, including Wnt and the PI3K/AKT/PTEN axis [38]. CK2 promotes survival through enhanced NF-κB activity to regulate the inflammatory response [39, 40], thus activating transcription of both IL-6 [41] and IL-8 [42], which are activated in the boa intestine (Fig. 3a-b). IL-8 Signaling is a central regulator with high overlap in expressed pathways at 1dpf and 3dpf (Fig. 2d), and both IL-8 and IL-6 signaling pathways are significantly active throughout the time series (Figs. 3a and 4a). Inhibition of PKA activity, observed in decreased activity of subunits PRKACB and PRKAR2B in the early post-feeding response, is associated with increased IL-6 signaling [43]. At this time, RAF1 activity may suppress JAK/STAT3 signaling, protecting from tumorigenesis in a high IL-6 and IL-8 signaling environment.

After the major axis of CK2-driven activity at 1dpf in IL-8, the regenerative response switches to suppress stress response pathways and allow proliferation and growth to increase. Although inhibition of eIF2 signaling in the early response is connected to increased UPR activity, the increasing inhibition of eIF2 signaling at 3dpf and 6dpf may in turn suppress the UPR [44]. Additionally, GSK3β activity changes at 12hrpf and 1dpf are consistent with downstream inhibitory effects on NRF2 stress response [45]. Finally, TLR signaling at 6dpf promotes late-phase NF-κB and Ap-1 expression to drive proliferation.

### Conserved regenerative signaling mechanisms across divergent snake models

**Fig. 5:**
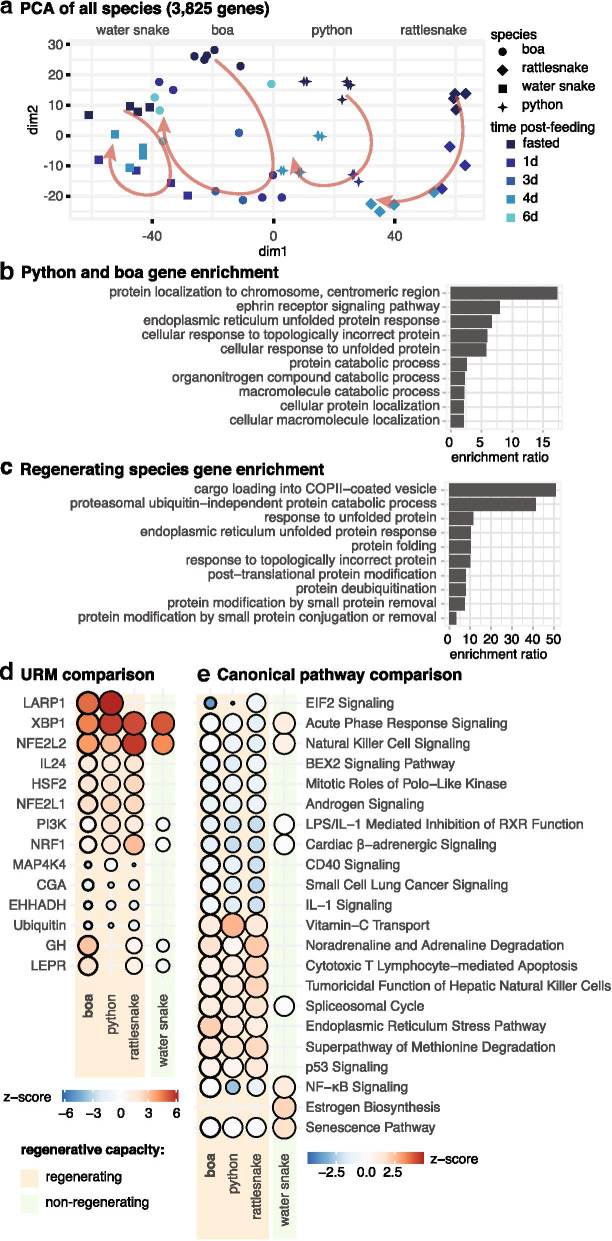
Comparison of responses associated with intestinal regeneration in multiple snake lineages. **a** Generalized PCA of orthologs with differential expression in a feeding contrast of at least one species. Arrows added to emphasize patterns. **b**, **c** GO enrichment of biological processes for genes that are uniquely differentially expressed in the boa and python and then in all three regenerating species. **d**, **e** Balloon plots of URM and canonical pathway activation highlighting major differences between regenerating species (*boa constrictor*, python, and rattlesnake) and a non-regenerating species (water snake)

To test whether multiple snake lineages with regenerative capacity utilize the same underlying mechanisms, we compared our results to those from a previous study that analyzed other snake lineages that do (pythons and rattlesnakes) and do not (water snakes) regenerate following feeding [18]. We evaluated responses to feeding across species using fasted vs. 1dpf RNAseq-based inferences of canonical pathway activation and URM activity. Across species, we find distinct cyclic temporal progressions of gene expression based on a generalized PCA, with clusters stratified by species indicative of species-specific nuances in gene expression (Fig. 5a). Among significantly differentially expressed genes between fasted and 1dpf for each species, boa and python share 285 genes in total, while over half of the genes differentially expressed in the boa are unique to that species (Fig. S16). Differences in both the temporal rate of the response [18] but in the number of RNAseq reads per species may contribute to some of this observed variation among species (Table S5). Genes uniquely expressed in boas and pythons function in metabolic processes, protein localization, and the UPR (Fig. 5b), and genes unique to all regenerating species are involved in COPII-vesicle transport and protein folding and modification (Fig. 5c).

Inferences of URM and canonical pathway activation highlight many signaling patterns that are shared among regenerating species with absent or opposing activation in the non-regenerating water snake. These results implicate a core set of pathways and molecules central to the onset (1dpf) of the regenerative response across species (Figs. 4b-c). Only the regenerating species show URM activation of IL24, HSF2, NFE2L1, and the PI3K family, and inhibition of MAP 4 K4 and ubiquitin, and the boa and python both demonstrated activation of LARP1 (Fig. 5d). Interestingly, the URMs XBP1 and NFE2L2 associated with UPR and NRF2 stress response pathways are highly activated in all species, while other stress response URMs are uniquely activated only in the three regenerating species (HSF2, NRF1, NRF2L) (Fig. 5d). CPA comparisons also indicate that all species significantly activate UPR and NRF2-Mediated Oxidative Stress Response, and the Superpathway of Cholesterol Biosynthesis (Fig. 5). Importantly, several pathways with shared activation patterns among regenerating species are distinct from the water snake and may represent conserved features of the snake regenerative response (Fig. 5e).

## Discussion

We present new evidence for a coordinated ‘switch’ in signaling activity derived from the interactions of nutrient uptake, stress, and growth pathways that direct the extreme regenerative response in snake small intestine (Fig. 4c). Our findings link well-characterized signaling mechanisms across a timeline of regenerative growth and identify key regulatory transcription factors and kinases, including novel interactions of these pathways, that drive distinct phases of this response. We further demonstrate that this model for intestinal regeneration appears to be conserved at the level of pathways and regulatory molecules across multiple snake lineages spanning at least 50MY of divergence. This regeneration is neither pathological nor in response to traumatic injury, in contrast to regeneration models from most vertebrate systems [10, 46], and exploits broadly conserved vertebrate pathways, such as mTOR signaling, the UPR, and TLR signaling. These findings raise the possibility that major components of the pathways underlying this response in snakes are conserved among vertebrates, providing the opportunity to explore these regenerative mechanisms and the roles of key regulatory molecules identified here to develop novel therapeutic targets and treatments to control intestinal regeneration.

Previous genomic analysis in Burmese pythons identified positive selection in a number of genes relevant to extreme regeneration [47], and transcriptional studies have vaguely implicated roles for growth and stress response pathways in this regenerative process in snakes, although the mechanistic details of these interactions have remained unclear [16, 17]. For example, consumption of large meals in snakes has been shown to result in in massive increases in blood plasma fatty acids, triglycerides, and other nutrients within the first 12 h post-feeding [48, 49], leading to subsequent massive shifts in oxidative metabolism [15, 50]. We combine diverse data types to generate new insight into how metabolic stress activates a multi-pronged stress response, and based on the actions of these pathways in other vertebrate systems [51–56], how they may suppress proliferative growth under the extreme stress conditions of early uptake and massive metabolic change following feeding. In particular, the addition of proteomic and phosphoproteomic data reveals key signals that are hidden in the transcriptional data to show how post-translational regulation and modification regulate regeneration, although the lower sensitivity of shotgun proteomics will only detect highly expressed proteins. Previous studies of vertebrate regenerative growth implicated the limiting role that elevated cellular stress, and its detrimental effects, impose on complex tissue regeneration in vertebrates [57, 58]. Our results expand our understanding of how snake systems achieve such extreme regenerative growth capacities through the early activation of pro-apoptotic and extensive stress responses pathways that temporarily inhibit pro-growth signaling until cellular stress can be mediated, thereby effectively escaping this constraint to achieve extreme tissue regeneration without severe injurious side-effects.

In addition to the central roles of major stress response pathways, inflammation is another key stress response with downstream effects on tissues that can be detrimental if not mediated, and links between inflammatory and regenerative responses are known from other examples of vertebrate regeneration [59, 60]. Wound repair and tissue regeneration relies on inflammatory responses to restore and rebuild tissue architecture, and the dynamics between pro-inflammatory and anti-inflammatory states are carefully moderated, possibly by important metabolic pathways [60]. In the boa, after the immediate oxidative and ER stress response, our results newly implicate the activation of a CK2/NF-κB/IL-6/IL-8 axis that controls inflammation and proliferation to support intestinal regeneration and the shift from stress response to growth. CK2 promotes cell survival through increased NF-κB activity to regulate the inflammatory response [39, 40]. This is further supported by the activation of two important NF-κB targets, IL-6 [41] and IL-8 [42], in the boa intestine, both of which promote inflammation, proliferation, and regeneration [61, 62]. Later in the regenerative response, our results indicate that TLR signaling promotes further proliferation and control of inflammation. While direct links between these pathways and metabolic pathways are unclear, the coupling of regeneration and massive metabolic change during snake intestinal regeneration provides an explicit model for understanding the relationship between inflammation and metabolism in tissue regeneration.

In some cases, we identify activation or inhibition of pathways that seem to conflict with our expectations. For example, loss of PTEN signaling leads to hyperproliferation, and we measure decreased PTEN early in the regenerative response when we predict low cell division while also finding simultaneous signaling events produce decreased proliferation. These results suggest that several complex networks interact to modulate early regeneration without tumorigenesis or other problematic growth under high stress conditions. Additionally, heterogeneity in the intestinal epithelium might also manifest in different cell populations that vary in stress levels and responses at a given time, resulting in some cells that continue division while others are extremely stressed or dying.

## Conclusions

Our new understanding of the molecular interactions that orchestrate the post-feeding regeneration response in the snake intestine highlights an intriguing and largely novel model that entails interactions between nutrient uptake, stress response and apoptosis, and growth pathways that direct tissue regeneration in a vertebrate model. This highly integrated molecular signaling model provides new valuable perspectives on how these pathways may be synergistically capable of directing regenerative capacity in other vertebrates, including humans, that naturally have limited capacity for regeneration [13], yet may possess all the conserved regulatory systems and interactions required for an analogous regenerative response. Investigating these signaling mechanisms in snakes and their conservation in other vertebrate systems present a promising way forward for understanding signaling mechanisms and pathway interactions that underlie organ regenerative growth in vertebrates.

## Methods

### Experimental design and animal care

Animal care and experimentation were conducted under an approved IACUC protocol at the University of Alabama (14-06-0075) in AAALAC-accredited facilities. Boa constrictors were obtained captive-bred from Strictly Reptiles (Hollywood, Fl, USA) and acclimated for at least 3 months in a standard rack housing system and 12:12 light cycle, with meals offered weekly. Before experimentation, they were fasted until in post-absorptive Phase III fasted states and then fed meals sized to approximately 25% of their body mass. Snakes were humanely euthanized by severing the spinal cord immediately behind the head at five time points: while fasted and then 12hrpf, 1dpf, 3dpf, and 6dpf (*n* = 5 per group; total *n* = 25) to provide a time series of the regenerative response. Sample sizes are consistent with previous experiments of snake regenerative physiology [16, 18, 50]. Snakes were randomized into experimental groups by body mass, with no consideration for sex because they were all juveniles. At the time of sampling, all snakes were juveniles with snout-vent length 116.1 ± 5.0 cm and body mass of 729.4 ± 84.0 g (mean ± SD) (Table S4), and all were included for physiological analyses. In the proximal small intestine for each snake, we measured: mucosa/submucosa width, enterocyte volume, maltase activity, aminopeptidase activity, and uptake rates for leucine, glucose, and proline. Wet masses of the esophagus, stomach, small intestine, and large intestine were measured, and samples were snap-frozen for downstream transcriptomic and proteomic use. Investigators were blind to group assignment during histology and physiological analysis.

### Tissue microscopy

We examined post-prandial changes in intestinal tissue thickness and enterocyte size and assessed ultrastructural changes to the intestinal brush border membrane using transmission electron microscopy following previous studies [16, 63]. We took 6 μm cross-sections of formalin-fixed, paraffin-embedded intestinal samples and stained them with hematoxylin and eosin on glass slides. Slides were imaged using a light microscope (Nikon Eclipse E100) with a custom camera by The Imaging Source linked to a computer loaded with Motic Image Plus software (Richmond, British Columbia, Canada). We took multiple measurements, including mucosal/submucosal thickness, muscularis/serosa thickness, and enterocyte height and width, from ten distinct locations per imaged section. We calculated enterocyte volume as the volume of a cylinder (volume = 0.5width^2^*π*height). Transmission electron microscopy was used to study ultrastructural changes to the intestinal brush border membrane. We fixed pieces of intestinal mucosa in 2.5% gluteraldehyde, postfixed them in 1% osmium tetroxide, dehydrated them in graded series of ethanol, and embedded them in Spurr’s epoxy resin. We cut ultra-thin sections (~ 80 nm) which were placed on copper mesh grids and viewed using a Hitachi (H-7650) transmission electron microscope at 20,000X magnification (resolution: 0.204 nm lattice, 0.36 nm point-to-point). We imaged sections of microvillus membrane with the scope’s high-definition (Orca) camera using Advanced Microscopy Techniques Corp. image analysis software.

### Nutrient uptake and enzyme activity measurement

We followed the everted-sleeve technique protocols from previous studies [16, 63] to measure brush-border uptake of multiple amino acids and glucose. Uptake was measured as total uptake (i.e., including both carrier-mediated and passive), for each of L-leucine and L-proline and carrier-mediated uptake for D-glucose as nmoles per min per mg of tissue [14, 64]. We calculated the total uptake capacity for each nutrient as the product of each mass-specific uptake rate and segment wet mass. To quantify intestinal enzyme activity, we assayed aminopeptidase (APN) following the Wojnarowska and Gray [65] procedure and maltase following the Dahlqvist [66] assay as previously described [63] from 1 cm segments of tissue as described in previous work [63]. We calculated the capacity for each enzyme as the product of tissue mass and mass-specific rates of activity (μmol per min per g tissue).

### Transcriptomic data generation and analysis

Twenty snakes were chosen for sequencing, from fasted (*n* = 4), 12hrpf (*n* = 5), 1dpf (n = 5), 3dpf (*n* = 3), and 6dpf (n = 3) timepoints (Table S4). We extracted total RNA from ~ 50 mg of snap-frozen tissue each with a Trizol Reagent (Invitrogen) protocol. Illumina mRNA-seq libraries for each sample were constructed using poly-A selection, RNA fragmentation, cDNA synthesis, and indexed Illumina adapter ligation via the TruSeq Stranded mRNA kit. After quantification with a BioAnalyzer (Agilent) and Qubit (Invitrogen), RNA libraries were pooled and sequenced on an Illumina NovaSeq using 100 bp paired-end reads.

Raw de-multiplexed RNAseq reads were filtered and trimmed using Trimmomatic v 0.32 [67]. We quantified gene expression counts using the *boa constrictor* transcripts [68] as a reference with Salmon v 0.14.1 [69] then imported the results to R with tximport v 1.10.1 [70]. Counts were normalized in DESeq2 v 1.22.2 [71], and pairwise tests for differential expression were done between consecutive time points (e.g., fasted vs. 12hrpf, 12hrpf vs. 1dpf) as well as all time points against the fasted, which represented a baseline (e.g., fasted vs. 12hrpf, fasted vs. 1dpf). We used IHW v 1.10.0 [72] for independent hypothesis weighting and filtered each comparison for genes with an IHW-adjusted *p*-value < 0.05 and |log2 fold change| ≥ 1.5. These results were analyzed with the core analysis function in Ingenuity Pathway Analysis (IPA) (Qiagen Inc.) to determine upstream regulatory molecule (URM) activation, canonical pathway expression, mechanistic networks, and predicted downstream effects on function.

Normalized counts were used in TCseq v 1.8.0 [73] to identify patterns of gene co-expression and assign strength of membership to genes in those co-expressed clusters, ranging from 0 (no membership) to 1 (perfect membership). For more in-depth functional enrichment analysis, we filtered clusters to only include genes with membership > 0.6. Gene lists for each cluster were analyzed for functional enrichment in the Cytoscape ClueGO+CluePedia apps [74–76] and filtered to false discovery rate < 0.05 using the Benjamini-Hochberg procedure. Functional enrichment of GO Biological Processes in the multiple species comparison were performed in WebGestalt [77] and filtered to *p* < 0.05. Because genes were annotated based on homology to human protein-coding genes, we used the entire set of human protein-coding genes as a background for enrichment analyses. To determine overlapping gene networks, we intersected expressed genes of all pathways at fasted vs. 12hrpf and filtered to only connections in which at least 50% of expressed genes in one of the pathways overlap. The network was manually annotated for growth and stress response pathway groupings. This was repeated for overlap of significant pathways for fasted vs. each other timepoint and filtered to 60% overlap.

### Proteomic and phosphoproteomic data generation and analysis

We extracted proteins from the small intestine of fasted (*n* = 4), 12hrpf (n = 4), 1dpf (n = 4), and 3dpf (*n* = 3) snakes with the T-PER Tissue Protein Extraction Reagent (Thermo Fisher, 78,510), using the same samples as were used for RNA extraction (Table S4). We purified proteins using the methanol-chloroform method and prepared samples for mass analysis according to previous protocols [18, 78].

To isolate phosphopeptides, extracted proteins in T-PER buffer were reduced in 50 mM dithiothreitol (DTT) at 56 °C for 30 min and then alkylated in 30 mM iodoacetamide at room temperature (~ 25 °C) for 30 min in dark condition. Samples were digested with proteolytic enzyme trypsin (enzyme-to-proteins ratio of 1:50 at 37 °C for 16 h). Digested peptides were desalted through C18 cartridges (ThermoFisher Scientific, IL, USA) and dried by Speed Vacuum [78]. The digested peptides dissolved in 0.1% formic acid were used for phosphopeptides enrichment using High-Select TiO_2_ Phosphopeptide Enrichment Kit (ThermoFisher Scientific, IL, USA) based on manufacturer instructions. Briefly, the column was activated using a washing and binding buffer. The digested peptides passed through an activated filter and again washed with binding buffer. After removing all excess buffer, enriched phosphopeptides were eluted with elution buffer (50 μL) twice. Enriched peptides were dried in a speed-vacuum and reconstituted with 0.1% formic acid in water for liquid chromatography-mass spectrometry (LC-MS/MS) analysis.

A Velos Pro Dual-Pressure Linear Ion Trap Mass Spectrometer (ThermoFisher Scientific, MA) coupled to an UltiMate 3000 UHPLC (ThermoFisher Scientific, MA) was used to analyze digested peptides and phosphopeptides as previously described [78]. We performed MS/MS data acquisition and processing with Xcalibur™ software v 2.2 (ThermoFisher Scientific, MA), and spectra were searched against species-specific protein databases generated from the *Boa constrictor* genome [68, 79] with Proteome Discoverer v 2.1 (ThermoFisher Scientific, MA). For proteomics data, SEQUEST search considerations and settings were as in previous data analysis to identify high confidence peptides [78]. For phosphoproteomics data, variable modifications were oxidation (methionine) and phosphopeptides (+ 79.966 Da; S; Serine /T; threonine/ Y; tyrosine) as dynamic modifications, and carbamidomethylation (peptides) of cysteine as the static modification. The cutoff of the false discovery rate (FDR) using a target-decoy strategy was less than 1% for both proteins and peptides. In addition, ptmRS [80] node is used in the consensus step of Proteome Discoverer to filter the true phosphopeptides. Other search settings matched the proteomics data [78].

For protein quantification, counts from peptide spectrum matches (PSM) were normalized, log2-transformed, and hierarchically clustered by expression patterns through the time series in R. These clusters were analyzed for functional and pathway enrichment analyses in WebGestalt, using the entire protein-coding gene set as a background [77]. Kinase enrichment was performed in KEA2 [81].

## Supplementary Information

**Additional file 1:** Supplementary figures S1-S17 detailing gene expression results.

**Additional file 2: Table S1.** Differentially expressed genes between timepoints in *boa constrictor* small intestine after feeding (*p* < 0.05, |log2FC| > 1.5).

**Additional file 3: Table S3.** Target molecules of NFE2L2 and their directional changes in the RNAseq dataset from fasted vs. 12hrpf.

**Additional file 4: Table S2.** Target molecules of XBP1 and their directional changes in the RNAseq dataset from fasted vs. 12hrpf.

**Additional file 5: Table S5.** Number of reads per sample in multiple species analysis.

**Additional file 6: Table S4.** Description of snakes sampled for analyses.

## Data Availability

The RNAseq datasets supporting the conclusions of this article are available in the NCBI Short Read Archive repository, PRJNA704392, SRP051827, and SRP200900. The shotgun proteomics datasets supporting the conclusions of this article are available in the ProteomeXchange Consortium via the PRIDE repository, PXD024327.

## Abbreviations

dpf: Days post-feeding
NRF2: Nuclear factor erythroid 2-related factor 2
UPR: Unfolded protein response
mTOR: Mechanistic target of rapamycin
PI3K: Phosphoinositide 3-kinase
AKT: Protein kinase B
hrpf: Hours post-feeding
APN: Aminopeptidase N
TCA: Tricarboxylic acid
URM: Upstream regulatory molecule
IPA: Ingenuity Pathway Analysis
mTORC2: mTOR complex 2
RICTOR: Rapamycin-insensitive companion of mammalian target of rapamycin
IGF-1: Insulin-like growth factor 1
JAK: Janus kinase
STAT: Signal transducer and activator of transcription
MEK1/2: Mitogen-activated protein kinase kinases 1 and 2
ERK1/2: Extracellular signal-regulated kinases 1 and 2
PPARα: Peroxisome proliferator-activated receptor alpha
RXRα: Retinoid X receptor alpha
PPAR: Peroxisome proliferator-activated receptor alpha
ERK5: Extracellular signal-regulated kinase 5
IL-8: Interleukin 8
IL-6: Interleukin 6
NF-κB: Nuclear factor kappa-light-chain-enhancer of activated B cells
AMPK: 5′ adenosine monophosphate-activated protein kinase
eIF2: Eukaryotic initiation factor 2
GSK3β: Glycogen synthase kinase 3 beta
ER: Endoplasmic reticulum
ATM: ATM serine/threonine kinase
BRCA1: Breast cancer type 1 susceptibility protein
PTEN: Phosphatase and tensin homolog
TGF-β: Transforming growth factor beta
p38 MAPK: p38 mitogen-activated protein kinase
ErbB: Erythroblastic oncogene B
EGF: Epidermal growth factor
ERK: Extracellular signal-regulated kinase
MAPK: Mitogen-activated protein kianse
PDGF: Platelet-derived growth factor
MEKK: Mitogen activate protein kinase kinase kinase
PRKC: Serine/threonine-protein kinase PrkC
XBP1: X-box binding protein 1
ATF4: Activating transcription factor 4
mTORC1: mTOR complex 1
NFE2L2: Nuclear factor, erythroid 2 like 2
ApoA1: Apolipoprotein A1
HSPA5: Heat shock protein family A member 5
BiP: Binding immunoglobulin protein
INSR: Insulin receptor
TFGB1: Transforming growth factor beta 1
MITF: Microphthalmia-associated transcription factor
ELK1: ETS transcription factor
GH: Growth hormone
RAF: Rapidly accelerated fibrosarcoma
TLR: Toll-like receptor
CSNK1E: Casein kinase 1 epsilon
CSNK2A2: Casein kinase 2 alpha 2
RPS6KA3: Ribosomal protein S6 kinase A3
CSNK2A1: Casein kinase 2 alpha 1
CDK2: Cyclin dependent kinase 2
CAMK2: Calcium/calmodulin-dependent protein kinase type II
PRKACB: Protein kinase CAMP-activated catalytic subunit beta
MLYK: Myosin light chain kinase
MKNK1: MAPK interacting serine/threonine kinase 1
SGK1: Serum/glucocorticoid regulated kinase 1
SRC: v-src sarcoma (Schmidt-Ruppin A-2) viral oncogene homolog
PRKAR2B: Protein kinase CAMP-dependent type II regulatory subunit beta
PKD2: Polycystin 2
CXCR4: C-X-C motif chemokine receptor 4
LPS: Lipopolysaccharide
PEDF: Pigment epithelium-derived factor
FLT3: Fms-like tyrosine kinase 3
UVC: Ultraviolet C
HGF: Hepatocyte growth factor
RAAS: Renin-angiotensin-aldosterone
CASP3: Caspase 3
CK2: Casein kinase 2
PKA: Protein kinase a
Ap-1: Activator protein 1
PCA: Principal component analysis
IL24: Interleukin 24
HSF2: Heat shock transcription factor 2
NFE2L1: Nuclear factor, erythroid 2 like 1
MAP 4K4: Mitogen-activated protein kinase kinase kinase kinase 4
LARP1: La-related protein 1
BEX2: Brain expressed X-linked 2
