# Supplementary material for: Identification of an integrated stress and growth response signaling switch that directs vertebrate intestinal regeneration
Source: BMC Genomics. 2022 Jan 4;23:6. doi: 10.1186/s12864-021-08226-5 (PMC8725436; doi:10.1186/s12864-021-08226-5)
Supplement: Supplementary file 1 — Additional file 1: Supplementary figures S1-S17 detailing gene expression results. [file 12864_2021_8226_MOESM1_ESM.docx]

Supplementary Materials for

**Identification of an integrated stress and growth response signaling switch that directs vertebrate intestinal regeneration**

Aundrea K. Westfall, Blair W. Perry, Abu H. M. Kamal, Nicole R. Hales, Madhab Sapkota, Drew R. Schield, Mark W. Pellegrino, Stephen M. Secor, Saiful M. Chowdhury, Todd A. Castoe^*^

*Corresponding author. Email: [todd.castoe@uta.edu](mailto:todd.castoe@uta.edu)

**This PDF file includes:**

Figs. S1 to S17

Tables S1 to S5


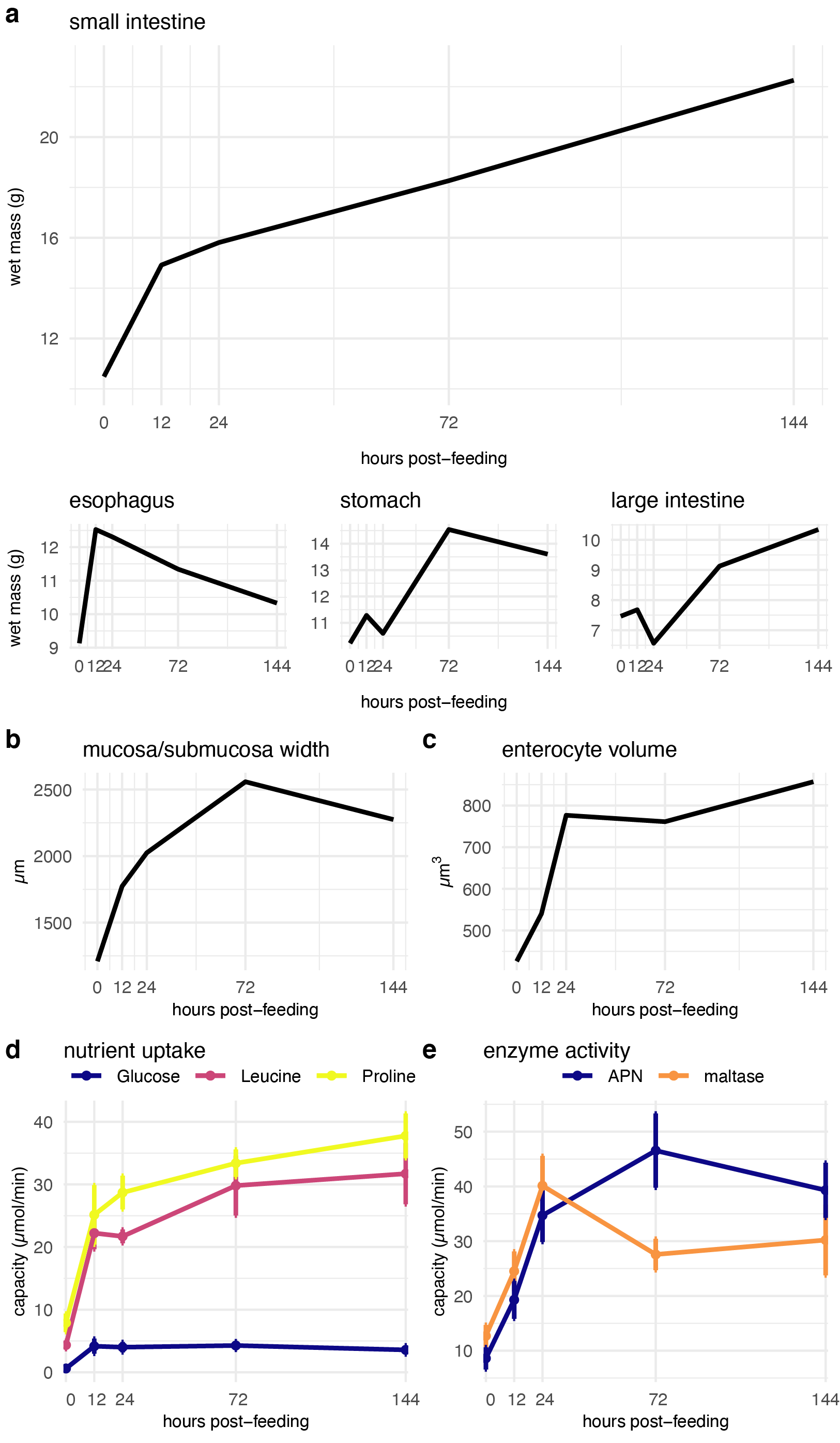


Fig. S1.

Mean measures of **(a)** various gastrointestinal tract organ wet masses, **(b)** mucosal tissue width, **(c)** enterocyte volume throughout the time series, **(d)** nutrient uptake capacity, and **(e)** enzyme activity capacity. Nutrient uptake and enzyme activity capacities include standard error bars.

**
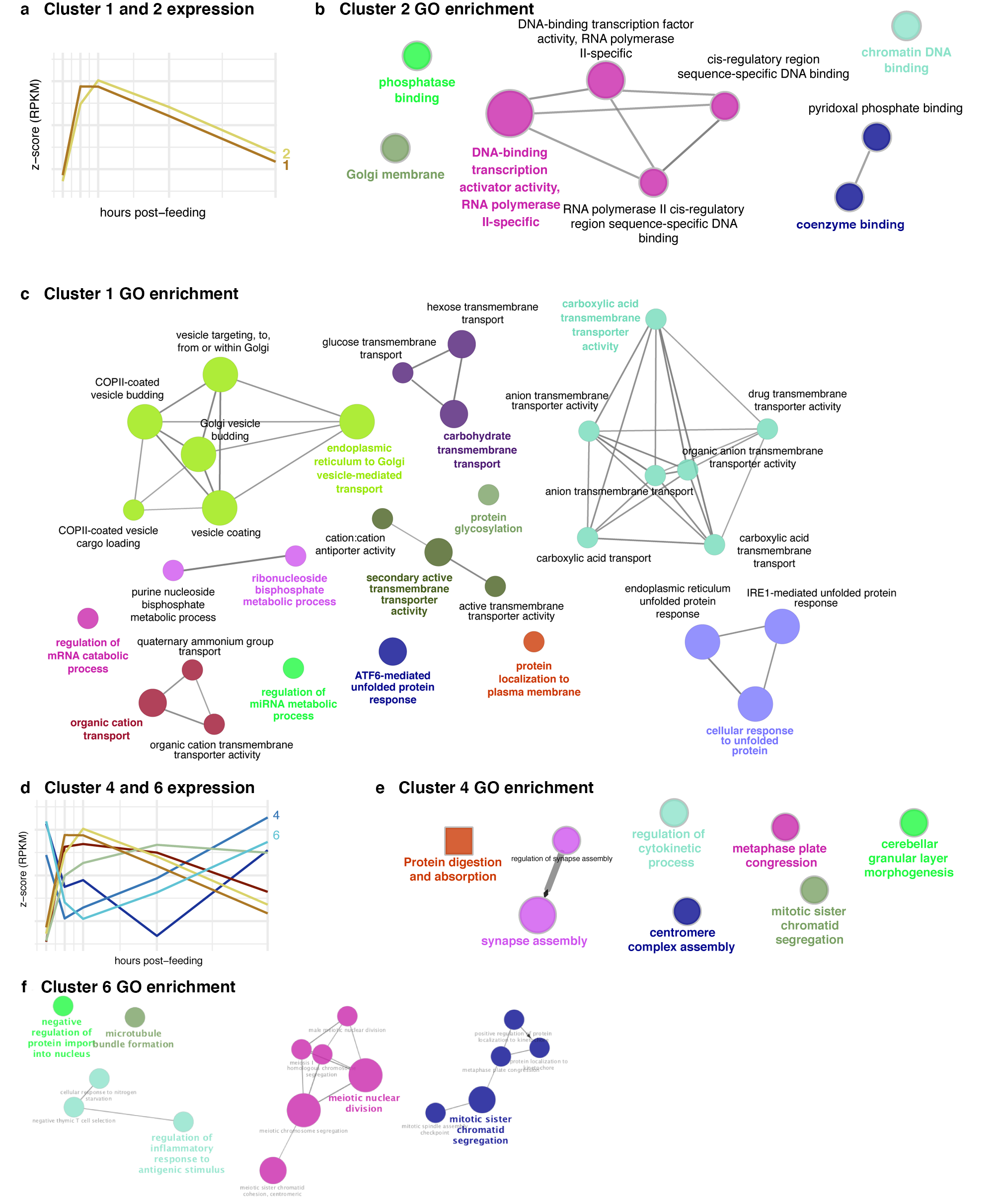
**

Fig. S2.

**(a)** Plot of expression patterns for RNAseq clusters 1 and 2, with GO enrichment terms for **(b)** cluster 2 and **(c)** cluster 1. **(d)** Plot of expression patterns for RNAseq clusters 4 and 6, with GO enrichment terms for **(e)** cluster 4 and **(f)** cluster 6.


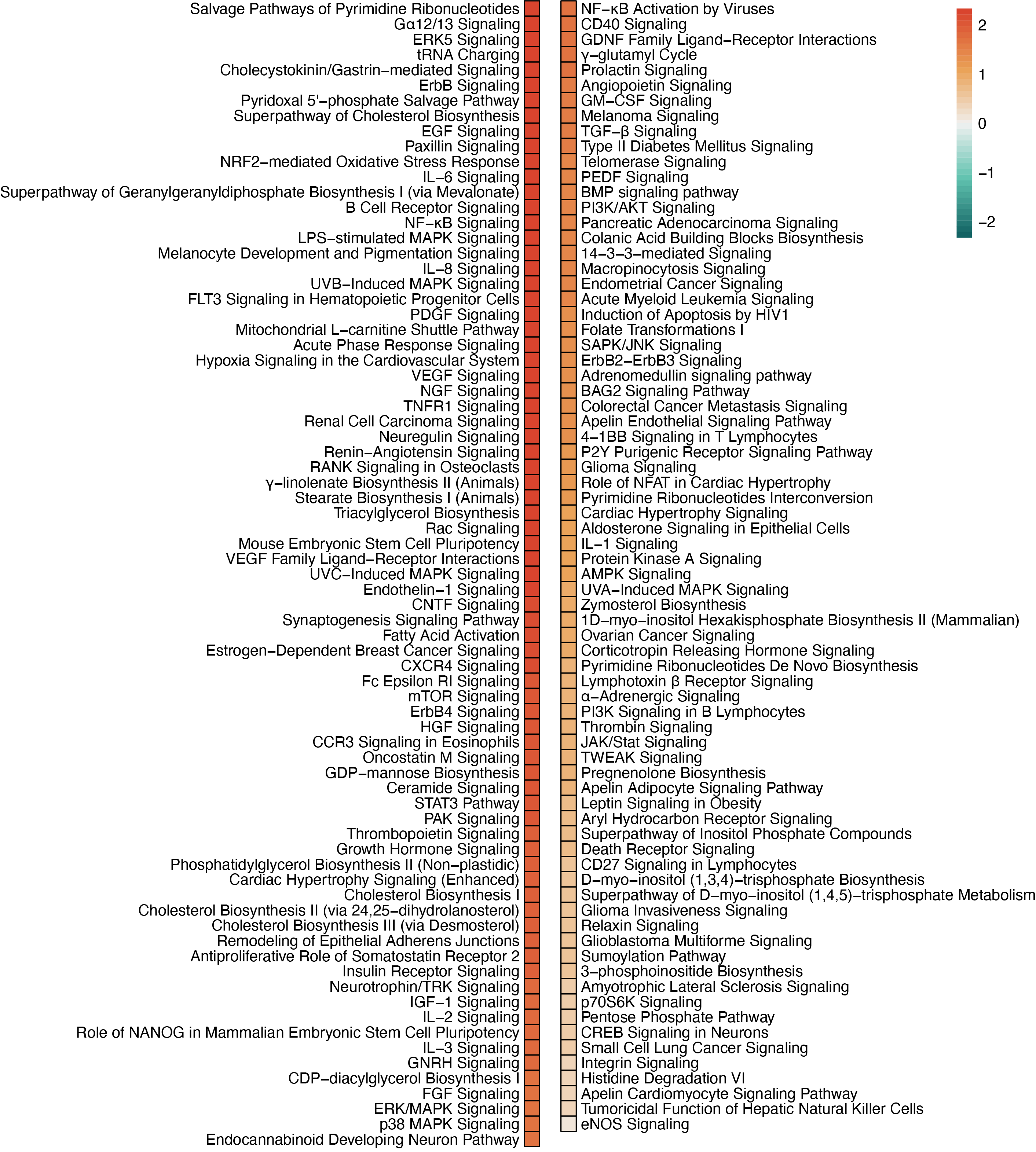


Fig. S3.

Heatmap of activated canonical pathways from fasted to 12hrpf.


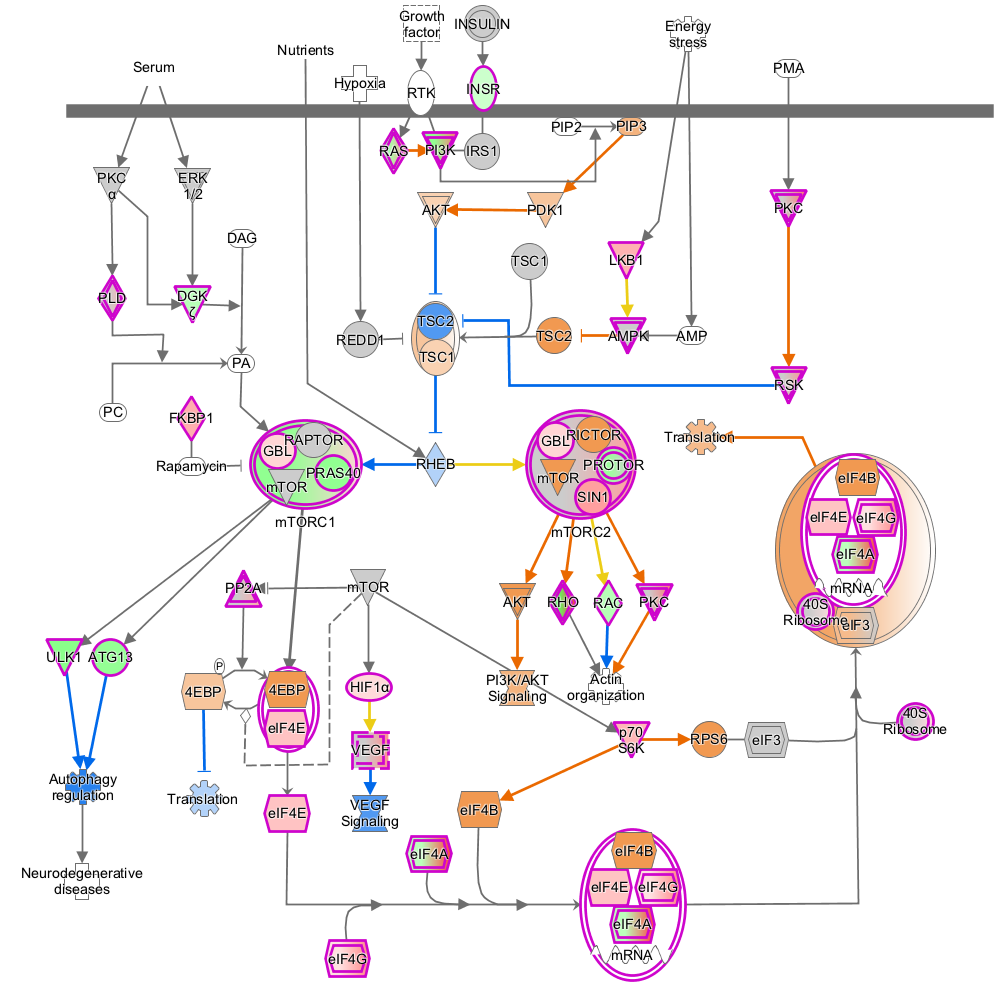


Fig. S4.

Molecule activity prediction of mTOR Signaling with the downstream upregulation of PI3K/AKT Signaling. Pathway activation indicated upregulation of the mTORC2 complex with RICTOR to promote increased PI3K/AKT signaling. The network figure was generated through the use of IPA (QIAGEN Inc., https://www.qiagenbioinformatics.com/products/ingenuity-pathway-analysis).

**
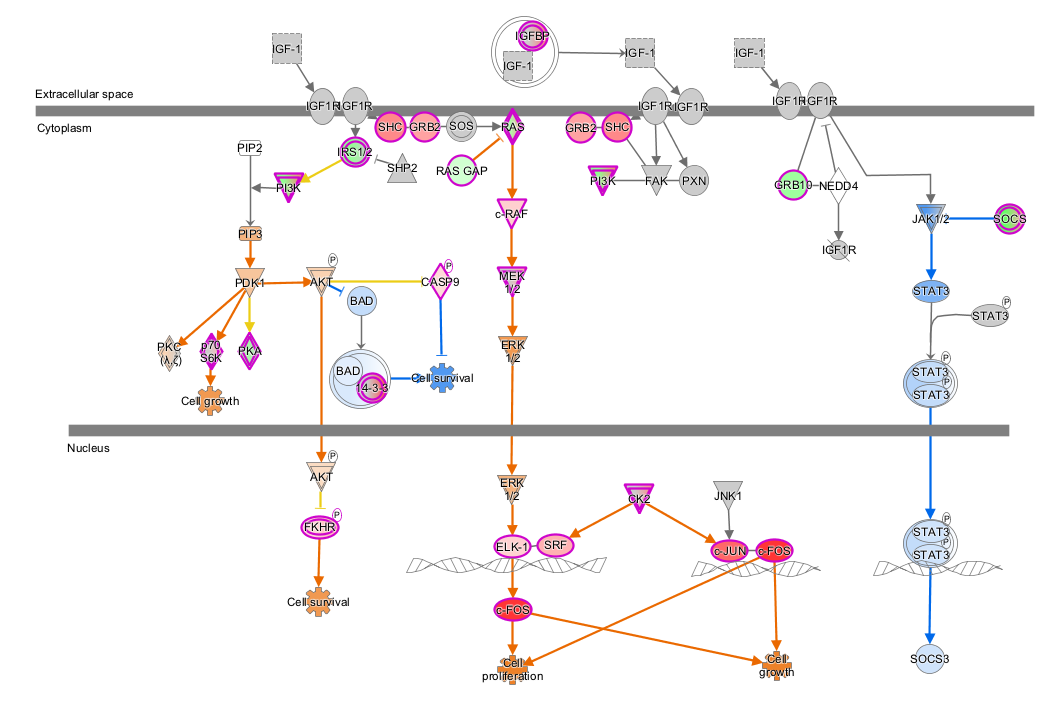
**

Fig. S5.

Molecule activity prediction of IGF-1 Signaling from IPA showing the downregulation of JAK/STAT signaling and activation of cell growth and proliferation via MEK1/2 and ERK1/2. Blue molecules are predicted downregulated, and green have decreased measurement in dataset. Orange are predicted upregulated, and red have decreased measurement. The network figure was generated through the use of IPA (QIAGEN Inc., https://www.qiagenbioinformatics.com/products/ingenuity-pathway-analysis).

**
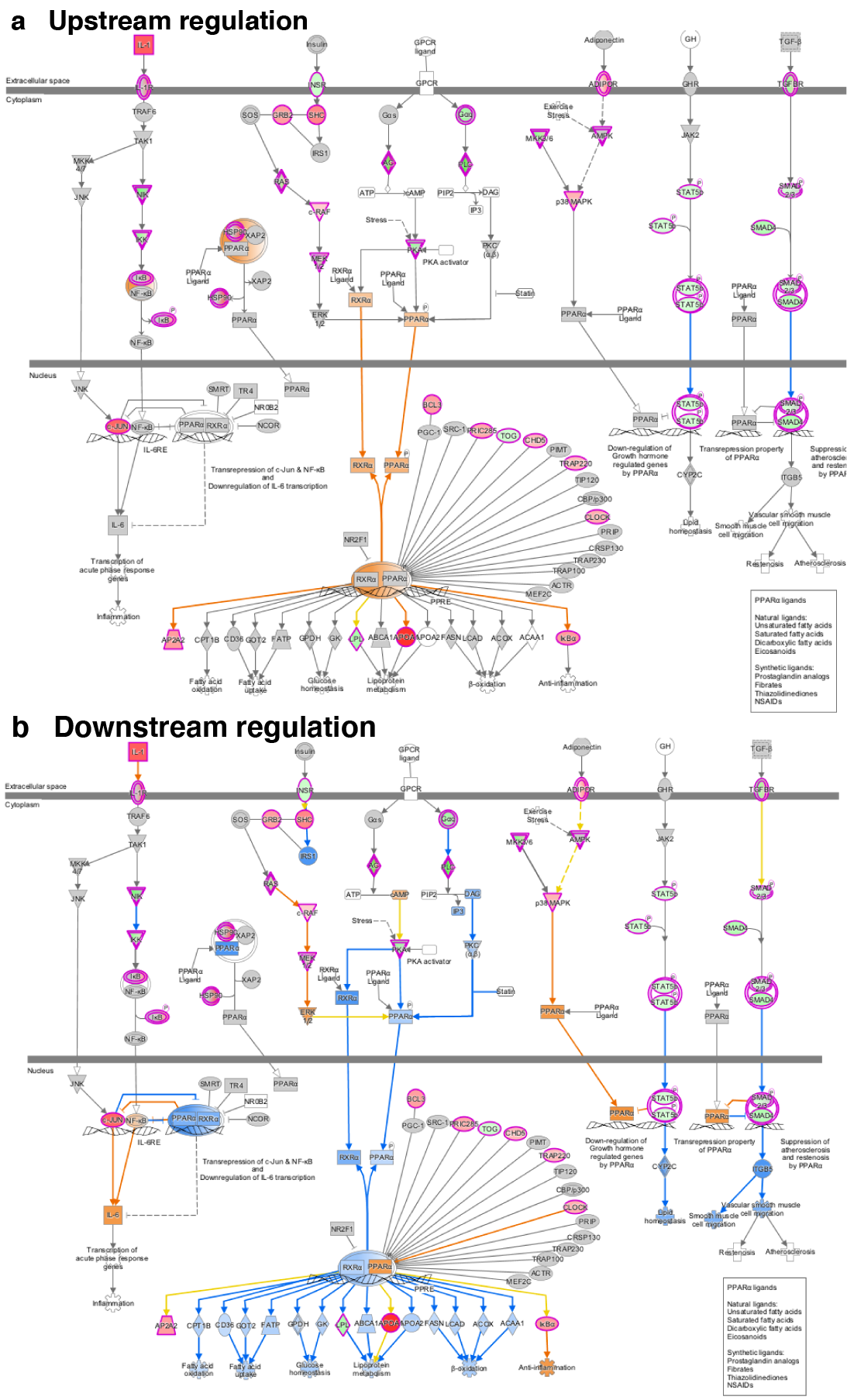
**

Fig. S6.

Molecule activity prediction of **(a)** up-stream and **(b)** down-stream signaling of PPARα/RXRα at fasted vs. 12hrpf, highlighting a feedback loop to decrease signaling. The network figures were generated through the use of IPA (QIAGEN Inc., https://www.qiagenbioinformatics.com/products/ingenuity-pathway-analysis).

**
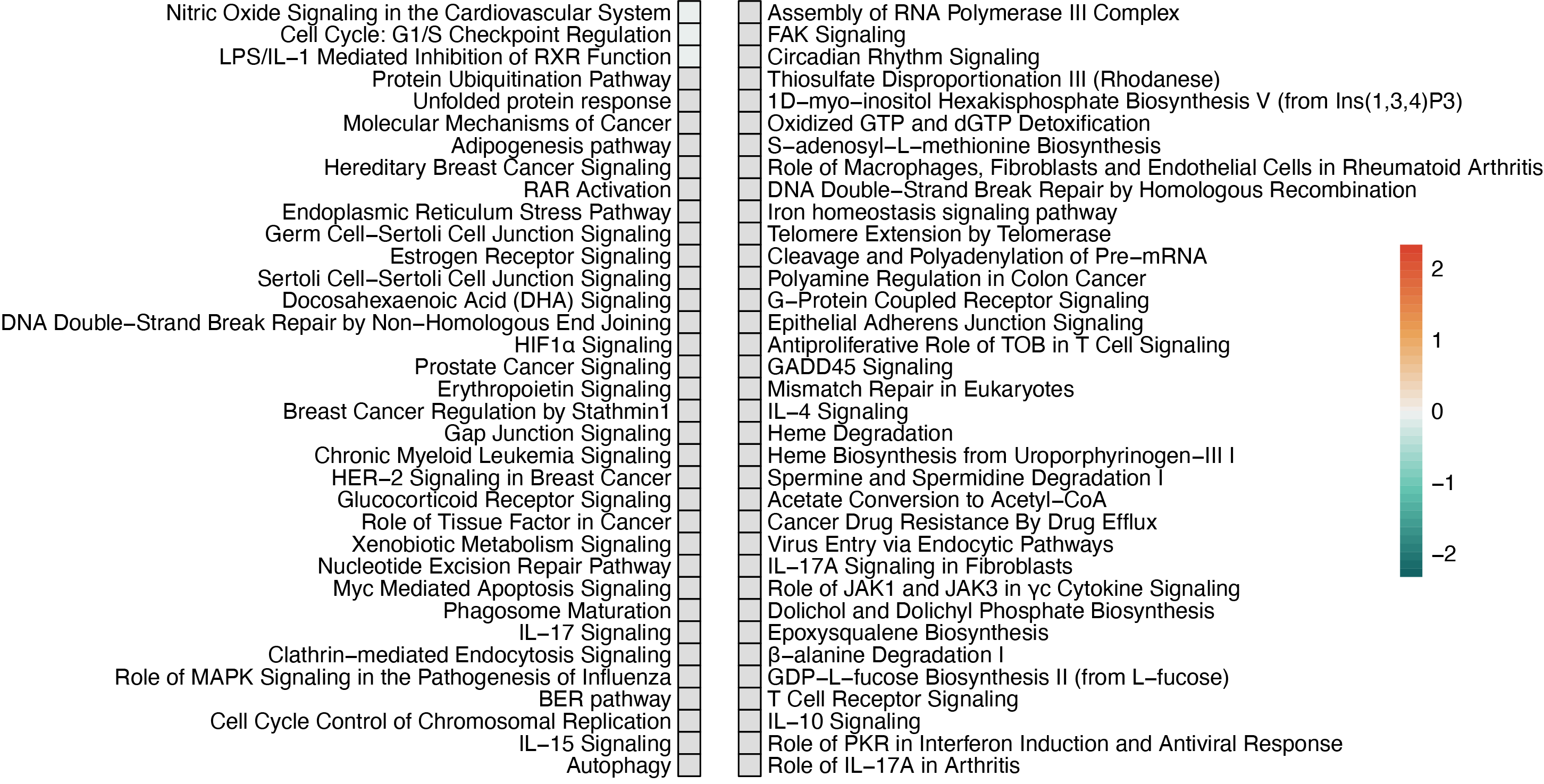
**

Fig. S7.

Heatmap of canonical pathways with significant presence but an activation score that cannot be calculated (i.e., z-score = NaN) or is not directional (i.e., z-score = 0) from fasted to 12hrpf.

**
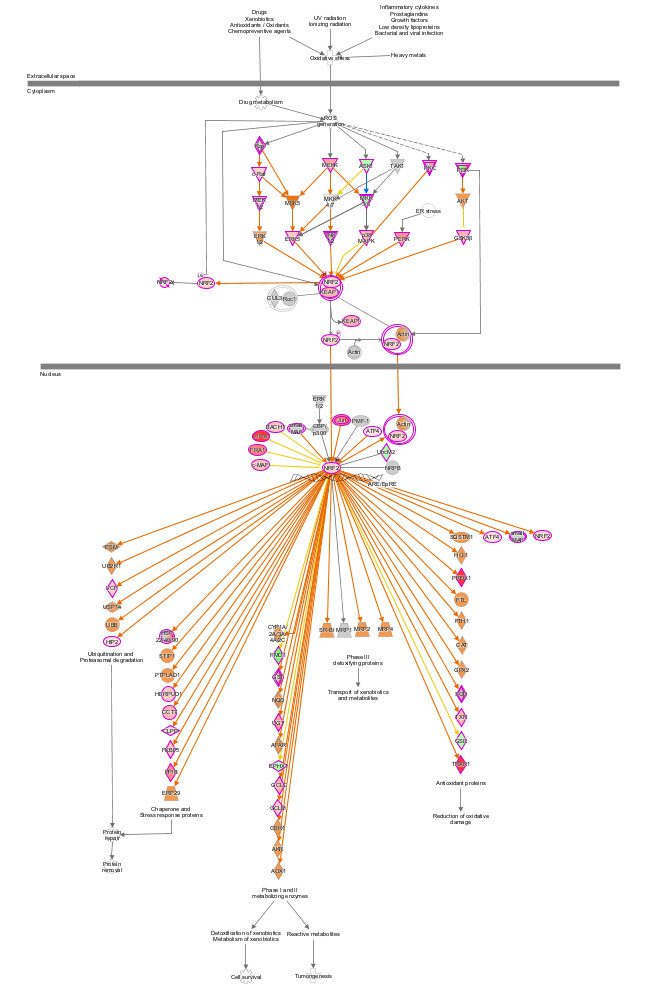
**

Fig. S8.

Molecule activity prediction of the NRF2-Mediated Oxidative Stress Response downstream of measured changes from fasted vs. 12hrpf. It is predicted to increase ubiquitination, proteasomal degradation, and chaperone and stress response proteins to repair and remove proteins; further increase antioxidant protein activity to reduce oxidative damage; and contribute to the formation and transport of metabolites. The network figure was generated through the use of IPA (QIAGEN Inc., https://www.qiagenbioinformatics.com/products/ingenuity-pathway-analysis).**
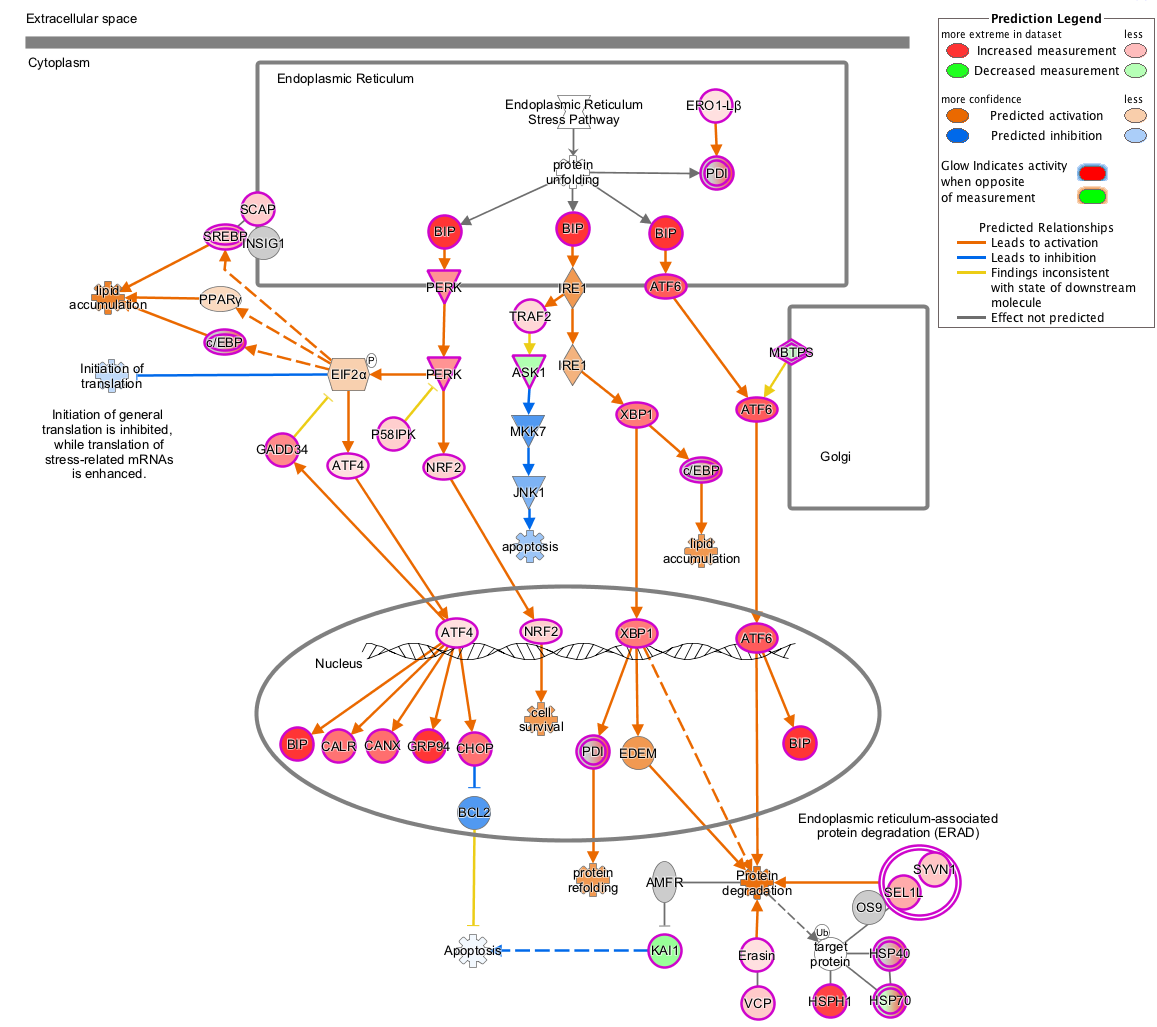
**

Fig. S9.

Molecule activity prediction of the Unfolded Protein Response downstream of measured changes from fasted vs. 12hrpf, highlighting increased lipid accumulation, protein degradation and refolding, and cell survival with decreased apoptosis. The network figure was generated through the use of IPA (QIAGEN Inc., https://www.qiagenbioinformatics.com/products/ingenuity-pathway-analysis).

**
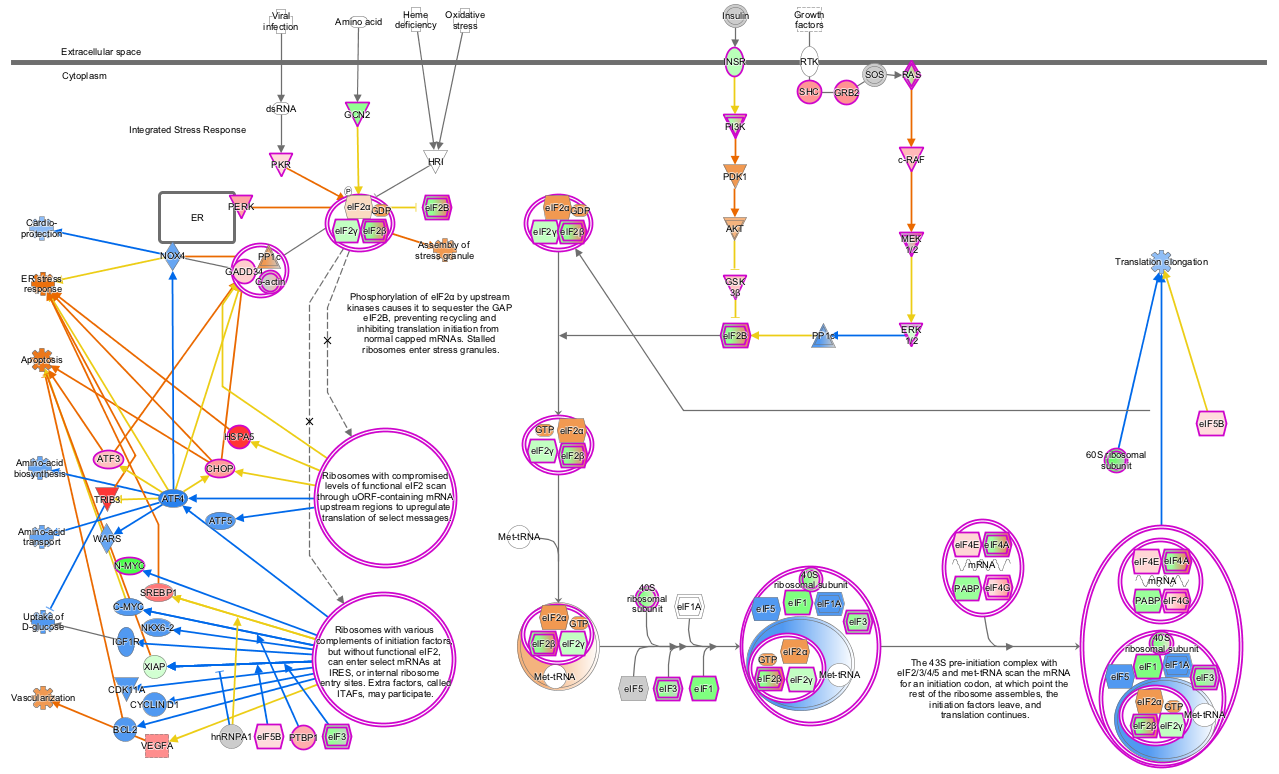
**

Fig. S10.

Molecule activity prediction of EIF2 signaling at fasted vs. 1dpf. eIF2 inhibition downstream of GSK3β activity leads to suppressed amino acid transport and D-glucose uptake and promotes apoptosis and ER stress response. The network figure was generated through the use of IPA (QIAGEN Inc., https://www.qiagenbioinformatics.com/products/ingenuity-pathway-analysis).**
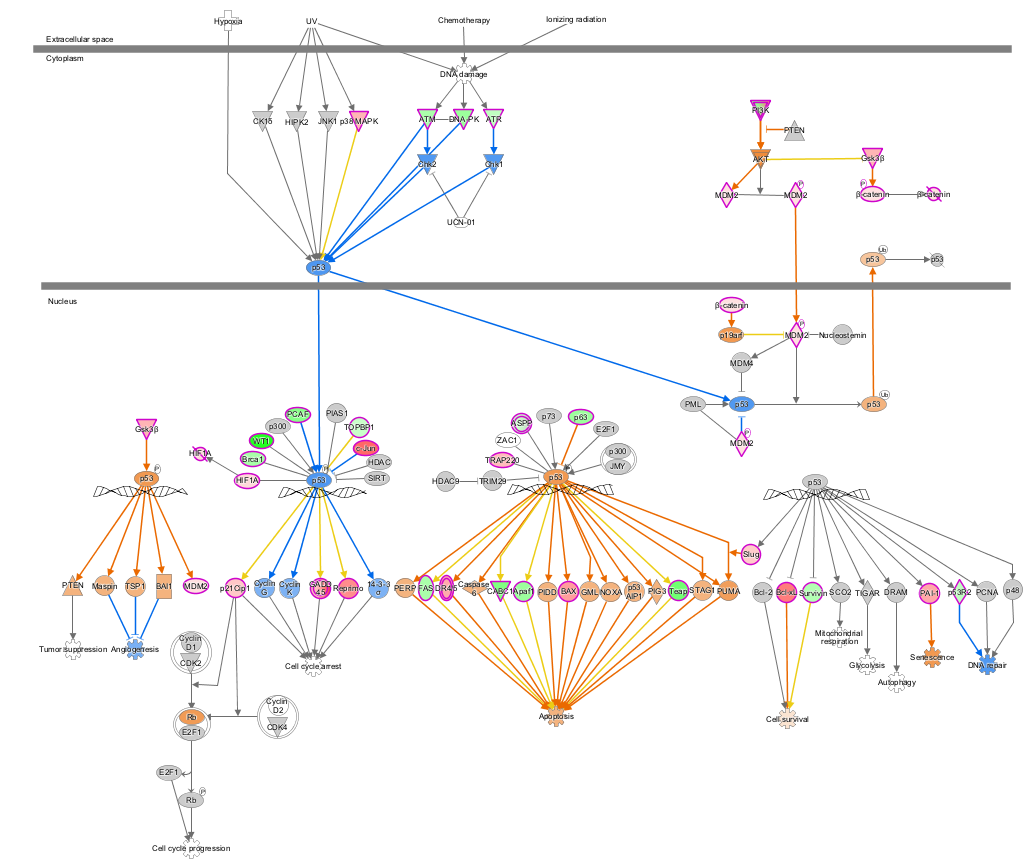
**

Fig. S11.

Molecule activity prediction of p53 Signaling at 12hrpf. The network figure was generated through the use of IPA (QIAGEN Inc., https://www.qiagenbioinformatics.com/products/ingenuity-pathway-analysis).


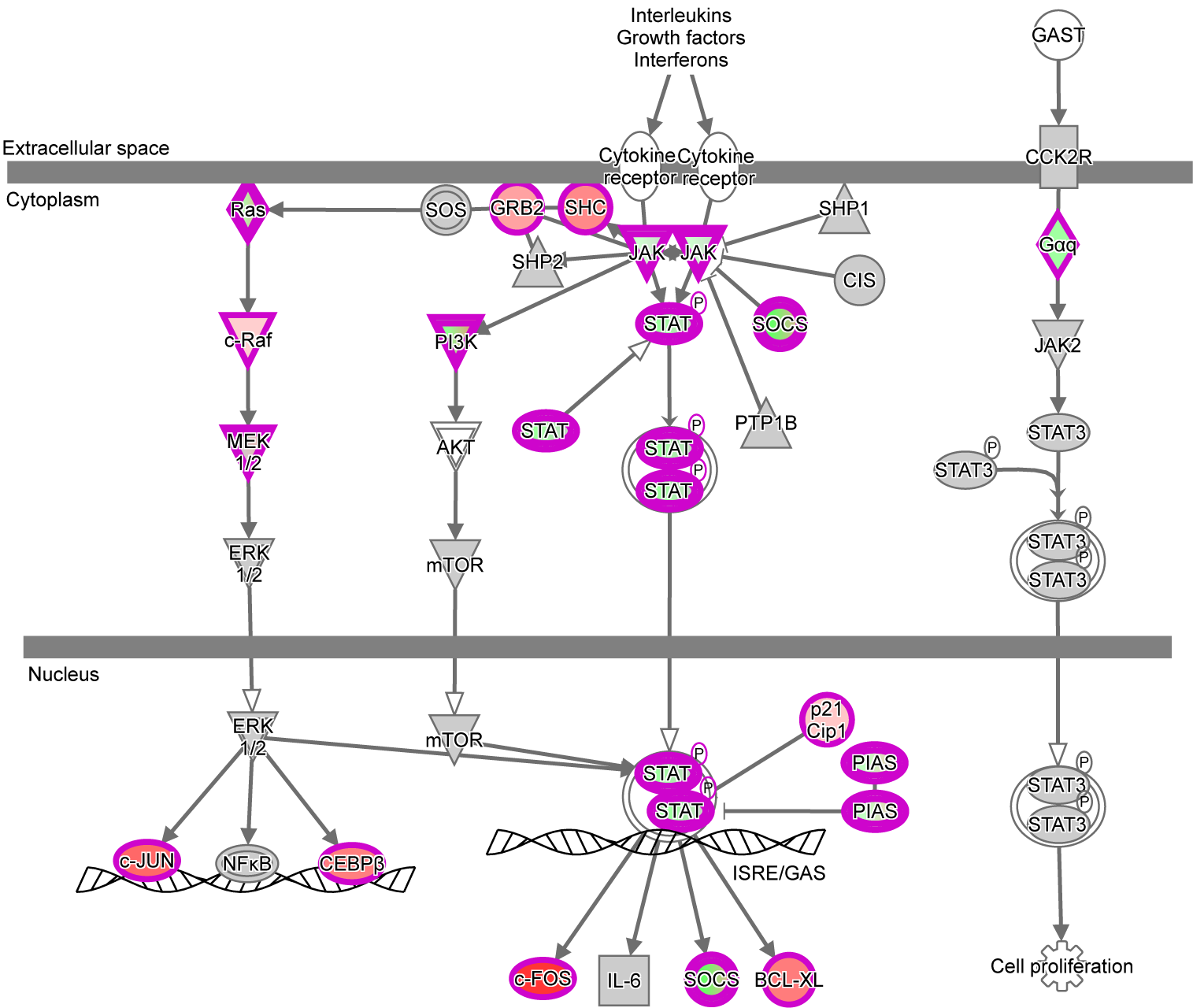


Fig. S12.

IPA molecule activity for the JAK/STAT signaling pathway, using expression changes from fasted to 12hrpf. Green shades indicate increased measurement, and red indicates decreased measurement. Although JAK/STAT signaling is predicted moderately active at 12hrpf (p-value < 0.01, z-score = 0.845), expression is restricted to a branch of the pathway that does not promote cell proliferation, and JAK and STAT proteins both have decreased measurement. The only expression in the proliferative branch is decreased measurement in a single gene early in the specific path. The network figure was generated through the use of IPA (QIAGEN Inc., https://www.qiagenbioinformatics.com/products/ingenuity-pathway-analysis).


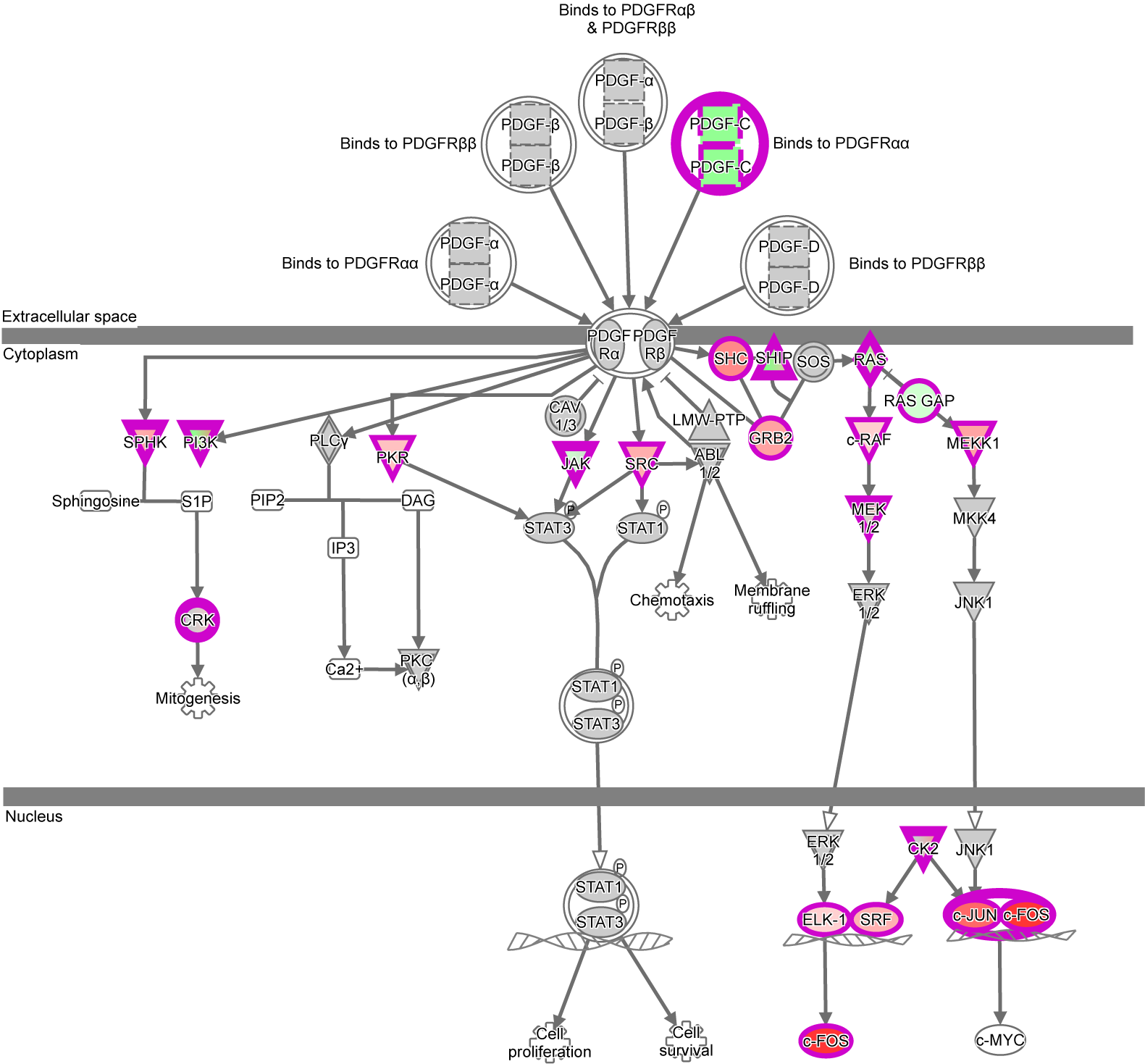


Fig. S13.

IPA molecule activity for the PDGF signaling pathway, using expression changes from fasted to 12hrpf. Green shades indicate increased measurement, and red indicates decreased measurement. Although PDGF signaling is predicted active at 12hrpf (p-value < 0.01, z-score = 2.548), expression patterns do not indicate that activity would promote cell proliferation at this stage of digestion. The network figure was generated through the use of IPA (QIAGEN Inc., https://www.qiagenbioinformatics.com/products/ingenuity-pathway-analysis).

**
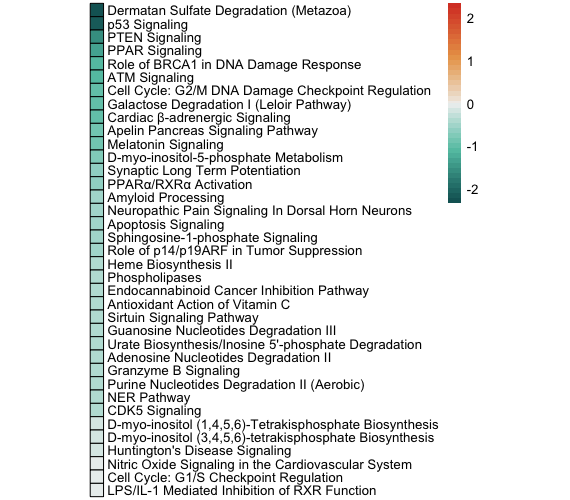
**

Fig. S14.

Heatmap of significantly inhibited (z-score < 0, p-value < 0.05) canonical pathways in IPA from fasted vs. 12hrpf gene expression contrasts.

**
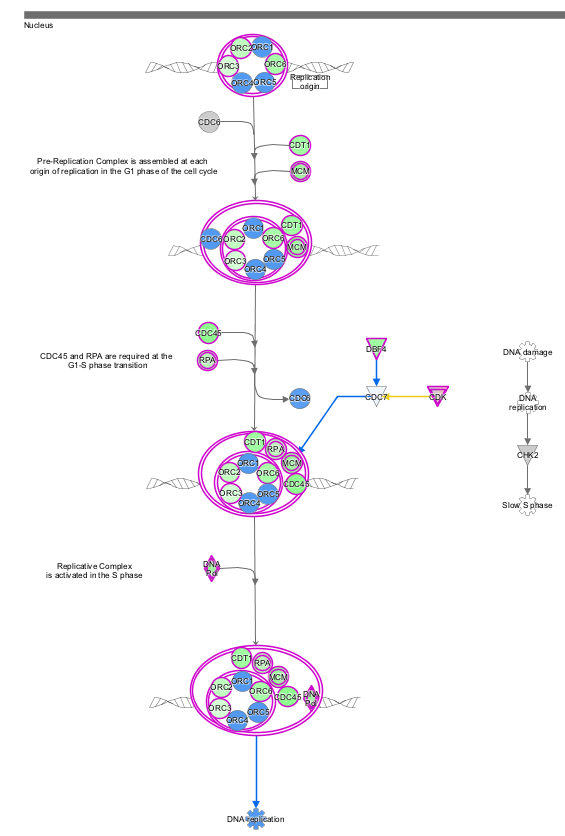
**

Fig. S15.

Molecule activity prediction of Cell Cycle Control of Chromosomal Replication at 12hrpf. The network figure was generated through the use of IPA (QIAGEN Inc., https://www.qiagenbioinformatics.com/products/ingenuity-pathway-analysis).


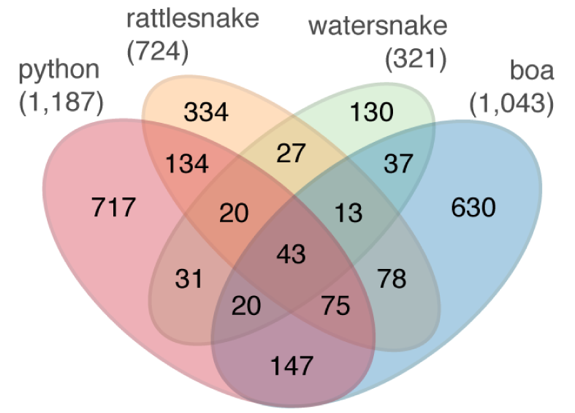


Fig. S16.

Venn diagram of overlapping homologs with differential expression in each species.

Table S1.

Differentially expressed genes between timepoints in boa constrictor small intestine after feeding (p < 0.05, |log2FC| > 1.5).

Table S2.

Target molecules of XBP1 and their directional changes in the RNAseq dataset from fasted vs. 12hrpf.

Table S3.

Target molecules of *NFE2L2* and their directional changes in the RNAseq dataset from fasted vs. 12hrpf.

Table S4.

Description of snakes sampled for analyses.

Table S5.

Number of reads per sample in multiple species analysis.
